# Inhibitory Control, Attentional Bias, and Palatable Food Consumption in Adolescents: A Laboratory Feasibility Randomized Controlled Trial

**DOI:** 10.2196/77579

**Published:** 2025-12-05

**Authors:** Carolina M Bejarano, Adrian Ortega, Christopher C Cushing

**Affiliations:** 1 Division of Behavioral Medicine and Clinical Psychology Cincinnati Children's Hospital Medical Center Cincinnati, OH United States; 2 Department of Pediatrics College of Medicine University of Cincinnati Cincinnati, OH United States; 3 Division of Developmental and Behavioral Health and Center for Children’s Healthy Lifestyle & Nutrition Children's Mercy Hospital Kansas City, MO United States; 4 Department of Pediatrics University of Missouri–Kansas City School of Medicine Kansas City, MO United States; 5 Department of Clinical Child Psychology & Schiefelbusch Institute for Life Span Studies University of Kansas Lawrence, KS United States

**Keywords:** hedonic appetite, adolescent, inhibitory control, attentional bias, palatable food

## Abstract

**Background:**

Understanding relationships among inhibitory control, attentional bias to food cues, and food consumption in nonclinical adolescent samples can inform preventive efforts for disordered eating and associated health risks.

**Objective:**

This pilot study conducted preliminary testing of an inhibitory control training intervention on attentional bias toward food cues, hedonic appetite, and food consumption, as well as examined inhibitory control’s relationship with hedonic appetite, food consumption, binge eating, and cognitive restraint.

**Methods:**

Participants (N*=*43; mean age 15.1, SD 1.7 years; 31/43, 72% female) were randomized to a food cue–specific go/no go inhibitory control training intervention or control group, took part in a laboratory “taste test,” and completed surveys and a dietary recall. Additional data were collected for subsamples through an eye tracking task (15/43, 35%) and cognitive tasks (19/43, 44%).

**Results:**

The go/no go intervention showed preliminary associations with attentional bias toward food cues. There were preliminary associations between inhibitory control and sugar consumption measured via dietary recall, binge eating symptoms, and cognitive restraint, which should be examined in a fully powered study.

**Conclusions:**

The role of inhibitory control should be examined in larger studies to inform strategies for promotion of healthy eating behavior and prevention of disordered eating in adolescents.

## Introduction

Reward sensitivity and inhibitory control have shown to be relevant factors in presentations of binge eating, although the most consistent findings come from adult samples in the context of studying obesity [1]. Few studies have examined these mechanisms simultaneously or in nonclinical adolescent samples to understand the risk of developing maladaptive eating patterns such as binge eating. There is existing evidence suggesting that difficulties with inhibitory control and higher reward sensitivity have both been associated with binge eating in youth [2], but further research is needed. The neurobehavioral inhibitory control model of feeding [3] proposes that inhibitory control, a dimension of executive function, interacts with hedonic appetite to influence palatable food consumption. In the context of dietary behavior, inhibitory control refers to inhibiting attention or a response toward a food cue [3]. In turn, hedonic appetite is a psychological and motivational process that refers to the drive to consume palatable foods for pleasure rather than for physiological sustenance [4]. In this model, attention allocation is thought to play a role in the dynamics between inhibitory control and hedonic appetite, with attentional bias to food cues referring to an increase in attention to food-related stimuli over other information [3,5]. A construct related to inhibitory control is cognitive restraint, which, in the context of dietary behavior, refers to efforts to avoid food intake. Cognitive restraint has been associated with higher cravings and pleasure derived from food [6].

Existing research has connected inhibitory control with hedonic appetite, palatable food consumption [7,8], dysregulated eating, and poor psychosocial outcomes in adults [9,10]. In adolescents, the literature has connected deficits in inhibitory control with overeating, risk of binge eating [11], palatable food consumption [12], negative affect [13], and hedonic appetite [14]. For example, functional magnetic resonance imaging data have indicated that adolescents with higher hedonic appetite have lower neural activation in the prefrontal cortex, suggesting that experiencing higher hedonic appetite may be related to lower inhibition of impulses to consume energy-dense palatable food [14]. In general, youth in the adolescent age group (13-18 years) have a higher tendency than other age groups to be high in reward seeking, vulnerable to developing disordered or maladaptive eating patterns, and at high risk of metabolic health concerns related to diet [15,16]. Moreover, adolescence is a key developmental stage to study drivers of dietary behavior as youth in this age group have increasing independence with their dietary choices and often consume a diet high in palatable foods that are lower in nutritional quality [17-19]. Therefore, adolescence presents a unique and important opportunity to understand and intervene on dietary processes that are carried into adulthood and impact long-term health.

Evidence-based methods used to study cognitive processes in the context of dietary behavior include reaction time paradigms to assess and intervene on inhibitory control [20] and eye tracking tasks to measure attentional bias toward food cues [21]. Food-specific inhibitory control training (ICT) tasks have been used to influence food choices [22]. A go/no go (GNG) ICT task is a procedure in which 2 categories of visual stimuli are randomly presented and a respondent is required to respond to images from one stimuli category (*go*) while withholding a response to an alternative stimuli category (*no go*) [23]. There is a majority of evidence suggesting that GNG training creates automatic stop associations that can influence outcomes of food choice, portion size, and weight changes [24] by training an individual to inhibit a response to food cues [22]. The few studies on GNG training in youth have found relationships with adolescents’ inhibitory control for intake of sugar-sweetened beverages [12] and reduction in consumption of energy-dense candy in children when measured in laboratory settings [25]. One pilot study found that a virtual reality ICT resulted in reduced binge eating up to the 2-week follow-up in adults [26]. While a GNG ICT task is often used as an intervention, a flanker task is an executive function measure that is used as an assessment of inhibitory control. The flanker task assesses a participant’s ability to allocate cognitive resources to process stimuli while also ignoring extraneous stimuli [27]. Finally, gaze fixation tasks with an eye tracker are often used to measure attentional bias toward food cues by measuring overall gaze direction and duration bias [28]. In adolescents, studies using gaze fixation with eye tracking data have found greater gaze duration bias toward food cues in a group with binge eating as compared to the control group [29].

The literature to date indicates that the field lacks studies that examine associations of inhibitory control in the context of dietary behavior in nonclinical samples of adolescents through both objective laboratory-based and subjective self-report outcomes [2]. An enhanced understanding of processes impacting dietary behavior in adolescents can be harnessed to inform preventive interventions geared toward improving health and well-being in this age group as well as in the long term into adulthood. The limited previous research to date has found that inhibitory control has been inversely related to facets of binge eating in community samples of adolescents and reward sensitivity has been related to binge eating behaviors in youth [2]. Together with the theoretical underpinnings of the neurobehavioral inhibitory control model of feeding [3] and the background literature described previously, this study was designed to examine the preliminary effects and pilot methodology of a randomized laboratory study of ICT and examine mechanisms of inhibitory control, attentional bias to food cues, hedonic appetite, palatable food consumption, and binge eating in a nonclinical sample of adolescents. The following hypotheses were tested: (1) the food-specific GNG task will show preliminary associations with reductions in attention to palatable food cues, hedonic appetite, palatable food consumption in a laboratory-based eating paradigm, and food intake via dietary recall; and (2) inhibitory control will show preliminary inverse associations with hedonic appetite, palatable food consumption (in a laboratory-based paradigm and via dietary recall), and binge eating and preliminary positive associations with cognitive restraint.

## Methods

### Participants

A total of 43 adolescent participants aged 13 to 18 years were recruited through the local community for a study about adolescent health behaviors. To best control for the food environment across participants and study dietary behavior in typically functioning adolescents, the inclusion criteria were living in a home setting with their parents or caregivers, the ability to read at their grade level and in English, and absence of blindness or low vision. The exclusion criteria were presence of developmental delays, past or current self-reported eating disorder diagnoses, and any health conditions or dietary restrictions that significantly affected activity or eating habits (eg, celiac disease or vegan diet).

### Procedure

#### Overview

The consent or assent process was completed at the laboratory visit, and it was communicated to parents or caregivers and adolescent participants that they were not fully informed of the processes being examined in the study but that they would receive more detailed information about the study after participating. Specifically, they were not informed about the study’s focus on dietary behavior or covert measurement of consumption through the bogus taste test until they completed the study. Adolescent participants also completed a survey indicating their palatable food preferences for the laboratory visit (ie, choosing one of each of the following: gummy bears or M&Ms in the sweet category, rolls or cereal in the starchy category, fried chicken or hot dogs in the fatty category, and potato chips and cheese pizza in the fast food category [30]). They were instructed to fast for 3 hours before the laboratory visit. The procedures described in this paper focus on the laboratory visit, which was the main component of a larger study that encompassed a 10-day protocol with evening surveys to assess within-person dietary processes [31]. Relevant to this study, on the 10th day, participants also completed a dietary recall.

#### Laboratory Visit

Participants were provided a standard meal replacement shake (350 calories with composition of 15% protein, 57% carbohydrates, and 29% fats [32]) at the beginning of the laboratory procedures in efforts to ensure that observed eating behavior was due to hedonic appetite rather than physiological hunger. Participants were then randomized to the intervention (food-specific) or control GNG task designed on the PsychoPy software (Open Science Tools Ltd) [33] using a computerized random assignment. Following the GNG task, procedures took place in the following order at the laboratory visit: completion of hedonic appetite self-report measure, bogus taste test, self-report survey battery, eye tracking task, Automated Self-Administered 24-Hour Dietary Assessment Tool (ASA24) dietary recall, National Institutes of Health (NIH) Toolbox cognitive tasks, and anthropometric measurements. The research assistant responsible for assessing the outcome in the bogus taste test remained masked to the randomization condition so as not to introduce bias in outcome measurement. The research assistant administering the GNG task and eye tracking task was aware of the randomization group to administer the designated task. All study procedures were conducted with scripted instructions used by the research staff to administer measures and tasks. The approach of administering the eye tracking and NIH Toolbox tasks to only a subset of participants was taken to pilot the protocols within the laboratory visit procedures.

#### ICT Task

**Figure 1:**
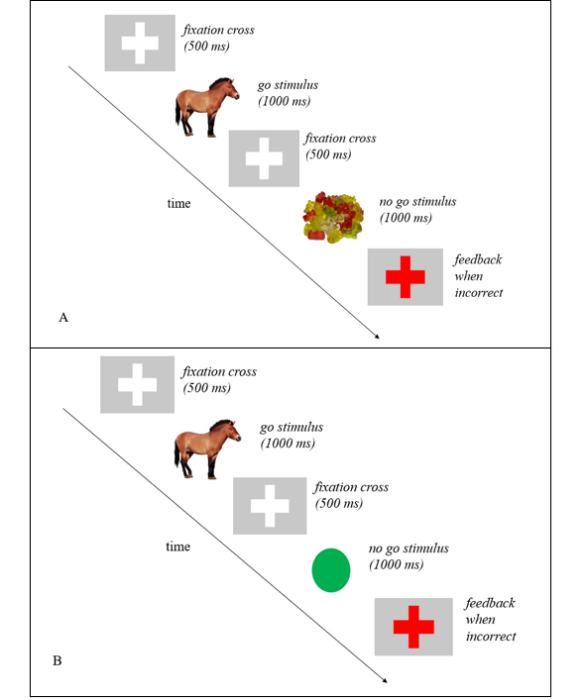
Examples of stimuli in the (A) food go/no go (GNG) task (intervention group) and (B) control GNG task.

The GNG procedures were designed in accordance with previous studies [25,34], including a practice round of the task to ensure that participants could perform it correctly. The GNG task included instructions to press a button when a *go* cue was presented and to not press a button when a *no go* cue was presented. Participants in the food-specific GNG intervention condition were trained using pictures of palatable foods grouped as sweet, starchy, fatty, and fast food as *no go* cues, with 4 food stimuli pictures used for each category (16 in total) and 16 nonfood stimuli pictures of animals as *go* cues. Those in the control GNG condition completed the GNG task with all nonfood stimuli (eg, animals and shapes; Figure 1). The images in the GNG tasks were used with approval from the Food-pics image database designed to facilitate standardization and comparability across studies [35] (Multimedia Appendix 1). For both groups, each stimulus was presented for 1000 ms, and the intertrial interval was 500 ms. If the participant made an incorrect response or did not respond within 1000 ms, a red cross appeared on the screen for 500 ms (Figure 1). For both groups, 50% of the trials were *go* trials (ie, 16 images of animals presented 4 times each), and 50% were *no go* trials.

#### Bogus Taste Test

After completing the GNG training, participants completed a hedonic appetite survey measure and then were brought to a separate room with a comfortable chair, an iPad playing a television show, and large amounts of the preferred palatable foods. Participants were allowed 20 minutes to complete the taste test and instructed to eat as much as they liked. The amount of each type of food eaten by each participant was weighed covertly by the masked research assistant to calculate the amount of palatable food consumed.

#### Eye Tracker and Remaining Tasks

A gaze fixation task with the use of an eye tracker was administered on a desktop computer to a subset of the study participants to determine attentional bias for food versus nonfood images. Participants then each completed self-report questionnaires, a dietary recall, and cognitive tasks and had their height and weight recorded. After the last day of study procedures, participants and parents were mailed their gift card compensation along with a debriefing letter outlining the research questions of interest.

### Constructs and Measures

#### Demographics and Anthropometrics

Participants completed a demographic questionnaire at the laboratory visit. Each participant’s height and weight were measured at the end of the laboratory visit following standard protocols. BMI percentile was calculated based on age and sex as indicated by the Centers for Disease Control and Prevention [36].

#### Food Preferences

The Food Preference Questionnaire was designed specifically for this study based on factor loadings from the Food Craving Inventory (ie, sweet, starchy, fatty, and fast food [30]) and on studies examining consumption of palatable food in a laboratory setting [7]. In this sample, the Food Craving Inventory demonstrated good internal consistency reliability (Cronbach α=0.93 for the total, 0.86 for sweet, 0.84 for starchy, 0.86 for fatty, and 0.76 for fast food). Participants completed this before the laboratory visit to determine which foods would be available during their bogus taste test.

#### Hedonic Appetite

The Power of Food Scale (PFS) is a 15-item measure that assesses the construct of hedonic appetite [37,38] using a 5-point Likert scale. Higher mean scores indicate higher hedonic appetite (range 1-5). The PFS has shown high internal consistency reliability in a previous sample of healthy adolescents (Cronbach α=0.94 [31]). The PFS was completed after completion of the GNG task.

#### Palatable Food Consumption

The amounts of the 4 foods offered in the bogus taste test were standardized based on caloric content. More specifically, research staff were trained to reliably measure and serve 500 to 600 calories of each food offered in the study. After the bogus taste test, the remaining portions of each food were weighed covertly on a food scale in grams, and the amount consumed was converted to calories.

#### Typical Dietary Intake

The ASA24 is a freely available web-based tool developed by the US National Cancer Institute [39] to obtain high-quality daily dietary data with minimal bias [40]. The 1-day dietary recall is considered acceptable as a valid assessment of typical dietary intake [40]. Two administrations of the ASA24 dietary recall took place: one at the laboratory visit and one 3 days after the laboratory visit.

#### Attentional Bias to Food Cues

Attentional bias was measured through administration of the gaze fixation task with the use of an eye tracker set up on a desktop computer. The amount of time participants spent looking at food versus nonfood images (referred to as dwell time bias or gaze duration bias) was used as a measure of attentional bias to food cues in the GNG intervention group as compared to the GNG control group. This measure of gaze fixation as attentional bias has shown excellent internal reliability and acceptable test-retest reliability in eye tracking procedures of this nature [41]. The eye tracking task was administered near the end of the laboratory visit procedures (ie, after the GNG task, bogus taste test, and laboratory visit surveys).

#### Eating Pathology

The Binge Eating and Cognitive Restraint subscales of the Eating Pathology Symptoms Inventory (EPSI) were used to measure disordered eating constructs of interest for this study. The EPSI is a well-validated 45-item self-report measure with 8 total subscales that has participants report on eating pathology in the previous 4 weeks [42] on a 5-point Likert scale. The binge eating subscale consists of 8 items, and scores range from 4 to 40. The cognitive restraint subscale measures cognitive efforts to limit or avoid eating and consists of 3 items, and scores range from 3 to 15. The binge eating subscale has shown excellent internal consistency in the sample of adolescents in this study [43], and the cognitive restraint subscale has shown good internal consistency reliability in a sample of adolescents previously [44].

#### Inhibitory Control

The NIH Toolbox iPad app provides access to the NIH Toolbox for Assessment of Neurological and Behavioral Function, a standard set of valid, reliable, and royalty-free tools for assessing cognitive, emotional, motor, and sensory function in participants aged 3 to 85 years. For this study, we used the NIH Toolbox Cognition: Executive Function model for assessing inhibitory control [45]. Participants completed a flanker task (Flanker Inhibitory Control and Attention Test for ages of ≥12 years; version 2.0) using the NIH Toolbox app on the iPad provided by the laboratory, which takes approximately 3 minutes to administer. The task has participants indicate the direction of an arrow that is flanked by 2 other arrows that point in either congruent or incongruent directions. The incongruent trials provide a measure of inhibitory control based on accuracy and speed, which is automatically calculated by the NIH program software. This task has shown to be a valid measure of inhibitory control and attention in youth [46].

### Data Analysis

#### Preliminary Analyses

Bivariate correlations and independent-sample 2-tailed *t* tests were run to examine associations between potential covariates (eg, eating disorder symptomology and BMI percentile) and the dependent variables. Significant correlations and associations were then examined further using independent-sample *t* tests to examine potential differences between the participants randomized to the food GNG intervention group and the control GNG group, with each potential covariate as a dependent variable. In particular, the dietary intake data obtained through the dietary recall were used as a randomization check to determine whether the 2 groups ate significantly different amounts of total calories over the 24-hour period before the study visit. Information regarding participants’ mental health and medication use that could potentially affect appetite and eating behavior ere also examined as potential covariates. Information was coded as having a potential influence on dietary behavior or not, and chi-square analyses were run to determine any differences between the randomization groups.

#### Effect of GNG Intervention

A series of independent-sample *t* tests were run to determine the effects of the GNG intervention versus the GNG control on gaze fixation on food images as measured using the eye tracker. Effects on hedonic appetite, palatable food consumption in the laboratory bogus taste test, and food consumption via dietary recall (second administration 3 days following the laboratory visit) were also examined.

#### Associations Between Inhibitory Control and Dietary Constructs

Regression models were run to examine cross-sectional relationships between inhibitory control as measured using the NIH Toolbox and the dietary constructs of interest (hedonic appetite, palatable food consumption in the laboratory, food consumption via dietary recall [first administration at the laboratory visit], and EPSI binge eating and cognitive restraint subscales).

### Ethical Considerations

All procedures followed in this study were in accordance with the ethical standards of the institutional review board, the 1964 Declaration of Helsinki and its later amendments or comparable ethical standards. This study was reviewed and approved by the institutional review board at the University of Kansas (STUDY00141427). Caregivers provided informed consent before data collection. No identifiable features of research participants are included in the data. Participants and caregivers were informed that participation was voluntary and they could discontinue at any time and were offered up to US $40 in compensation and snacks.

## Results

### Descriptive Information

**Figure 2:**
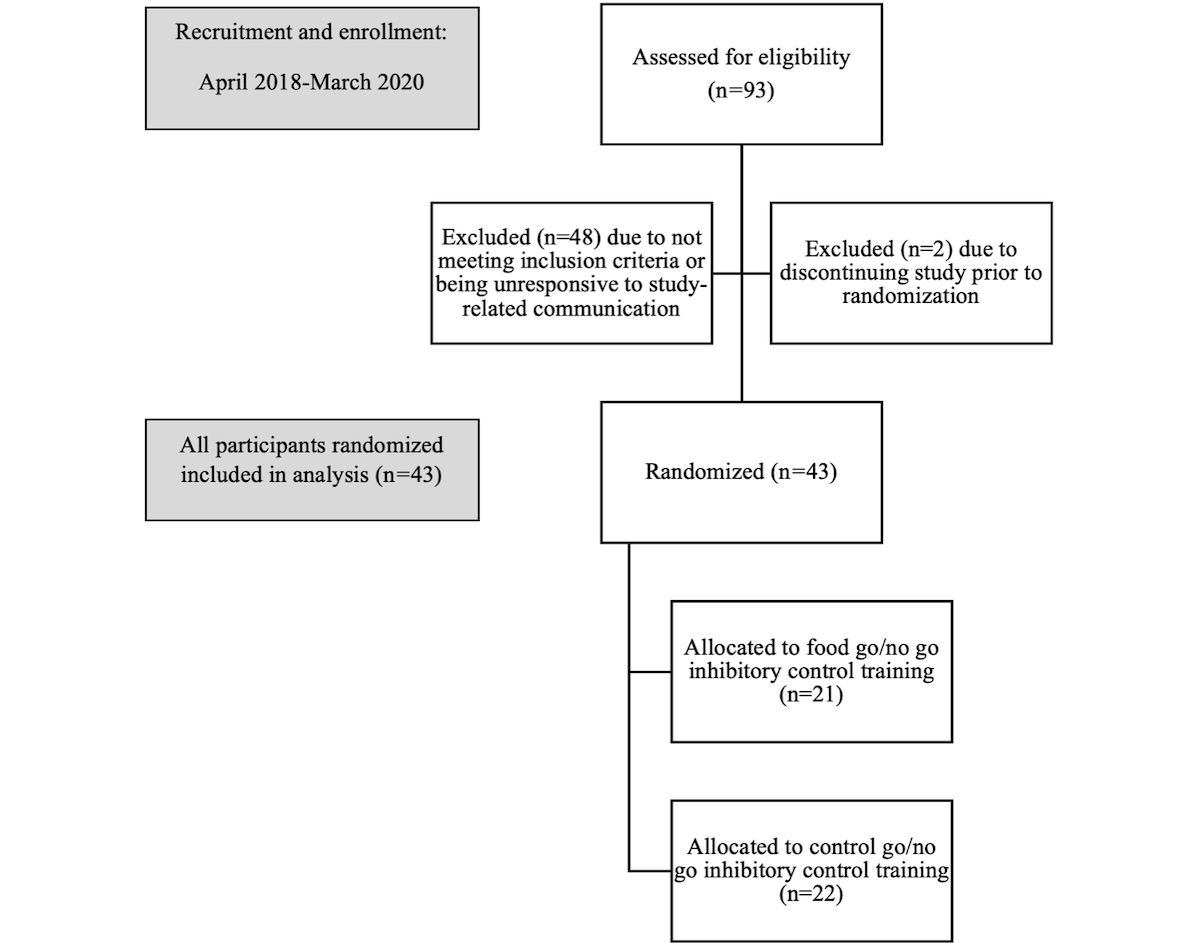
Flowchart of participant inclusion.

A total of 43 participants were enrolled in the study. Table 1 shows demographic information and descriptive statistics, and Figure 2 shows a flowchart of participant inclusion. The subset of participants who completed the gaze fixation task consisted of 15 adolescents (n=6, 40% from the GNG intervention group and n=9, 60% from the control group), and the subset of participants who completed the NIH Toolbox task consisted of 19 adolescents (n=10, 53% from the intervention group and n=9, 47% from the control group). Participants had a mean age of 15.1 (SD 1.7) years, most were female (n=31, 72%) and White (n=25, 58%), and 49% (21/43) reported an annual household income of >US $60,000. The average BMI percentile was 50.2 (SD 28.7), and the average calories consumed the day before the study as measured through the dietary recall was 1798.7 (SD 672.3). Additional data about adolescent mental health and medication history were obtained from 81% (35/43) of the participant-parent dyads. Among them, 17% (6/35) reported current or past history of anxiety, 11% (4/35) reported current or past history of depression, 14% (5/35) reported current or past history of attention-deficit/hyperactivity disorder, 6% (2/35) reported current or past history of other concerns, and 0% reported current or past history of eating disorders. The same dyads reported current medications for the adolescents: 11% (4/35) reported taking medication for anxiety or depression, 14% (5/35) reported taking acne medication, 9% (3/35) reported taking attention-deficit/hyperactivity disorder medication, 3% (1/35) reported taking allergy medication, 3% (1/35) reported taking thyroid medication, and 9% (3/35) reported taking other unspecified medication. For the taste test, in the sweet category, 54% (23/43) of the participants chose M&Ms, and 47% (20/43) chose gummy bears; in the starchy category, 47% (20/43) chose cereal, and 54% (23/43) chose rolls; in the fatty category, 81% (35/43) chose fried chicken, and 19% (8/43) chose hot dogs; and, in the fast food category, 30% (13/43) chose potato chips, and 70% (30/43) chose pizza. The average consumption in the laboratory taste test was 91.9 (SD 72.1) sweet calories, 79.9 (SD 95.6) starchy calories, 140.3 (SD 99.5) fatty calories, 178.9 (SD 143.5) fast food calories, and 491.0 (SD 249.9) total calories. The results of the randomization check indicated that the control group consumed an average of 1742.9 (SD 724.2) calories and the intervention group consumed an average of 1831.2 (SD 633.8) calories in the first administration of the dietary recall (*P*=.79; nonsignificant difference). The results of the chi-square analyses indicated that there were no substantial differences between the randomized groups and mental health conditions or medication use that could influence dietary behavior. The results of covariate screening indicated that the randomization process functioned effectively in controlling for any demographic variables associated with the dependent variables.

**Table 1:** Demographic information and descriptive statistics (N=43)^a^.

Demographic variable		Total	Intervention (n=21)	Control (n=22)
**Sex, n/N (%)**				
	Female	31/43 (72)	15/21 (71)	16/22 (73)
	Male	12/43 (28)	6/21 (29)	6/22 (27)
**Race or ethnicity, n/N (%)**				
	Asian	8/43 (19)	5/21 (24)	3/22 (14)
	Biracial or multiracial	4/43 (9)	3/21 (14)	1/22 (5)
	Hispanic or Latino	4/43 (9)	1/21 (5)	3/22 (14)
	White	25/43 (58)	11/21 (52)	14/22 (64)
	Other or did not report	2/43 (5)	1/21 (5)	1/22 (5)
**Approximate annual family income (US $), n/N (%)**				
	<30,000	11/39 (28)	6/19 (32)	5/20 (25)
	30,000-60,000	7/39 (18)	2/19 (11)	5/20 (25)
	>60,000	21/39 (54)	11/19 (58)	10/20 (50)
Age (y), mean (SD)		15.1 (2)	15.0 (1.6)	15.2 (2)
BMI percentile^b^, mean (SD)		50.2 (29)	52.6 (30.8)	47.7 (27.0)
Calories consumed in dietary recall 1, mean (SD)		1798.7 (672.3)	1831.2 (633.8)	1742.9 (724.2)
Calories consumed in dietary recall 2, mean (SD)		1551.5 (697.9)	1650.9 (746.1)	1452.0 (652.2)
Sugar calories consumed in dietary recall 1, mean (SD)		99.8 (64.7)	108.9 (60.6)	91.2 (68.9)
Sugar calories consumed in dietary recall 2, mean (SD)		72.6 (39.0)	78.5 (41.6)	66.8 (36.5)
Hedonic appetite (PFS^c^ score; 1-3.9), mean (SD)		1.6 (0.69)	1.6 (0.74)	1.6 (0.66)
Binge eating (EPSI^d^ score; 4-40), mean (SD)		18.4 (8.0)	19.3 (8.8)	17.5 (7.3)
Cognitive restraint (EPSI score; 3-15), mean (SD)		7.0 (2.9)	6.9 (2.1)	7.1 (3.6)
Inhibitory control (NIH^e^ Toolbox), mean (SD)		83.6 (10.7)	79.2 (8.8)	87.6 (11.1)
Sweet calories consumed (laboratory), mean (SD)		91.9 (72.1)	82.4 (79.2)	101.0 (65.2)
Starchy calories consumed (laboratory), mean (SD)		79.9 (95.6)	102.8 (110.4)	58.1 (75.1)
Fatty calories consumed (laboratory), mean (SD)		140.3 (99.5)	140.9 (88.8)	139.8 (110.8)
Fast food calories consumed (laboratory), mean (SD)		178.9 (143.5)	161.5 (132.5)	195.4 (154.5)
Total calories consumed (laboratory), mean (SD)		491.0 (249.9)	487.6 (247.8)	494.3 (257.8)

^a^A total of 84% (36/43) of the participants completed dietary recalls at both time points, and 9% (4/43) of the participants did not report approximate family income.

^b^In total, 85% (34/40) of the sample had a BMI percentile within the range categorized as normal weight, 10% (4/40) had a BMI percentile in the range categorized as overweight, and 5% (2/40) had a BMI percentile in the range categorized as obesity. BMI percentile data were missing for 3 participants.

^c^PFS: Power of Food Scale.

^d^EPSI: Eating Pathology Symptoms Inventory.

^e^NIH: National Institutes of Health. A total of 19 adolescents (n=9, 47% from the intervention group and n=10, 53% from the control group) completed the NIH Toolbox measurement of inhibitory control (age-corrected standard score for inhibitory control range 63-111).

### Results of the GNG Intervention

In the small subsample of participants who completed the gaze fixation task with eye tracking (15/43, 35%), participants from the intervention condition looked at food images for 20.85 (SD 10.25) seconds and participants from the control group looked at food images for 23.01 (SD 8.39) seconds. Therefore, the participants from the intervention condition looked at food images for 2.1 seconds less than control participants in absolute terms (95% CI −8.3 to 12.6). Expressed as an effect size, this is considered a small effect (Cohen *d*=0.23). There were no other significant differences in hedonic appetite or food consumption via the bogus taste test or dietary recall following the laboratory visit. Given the limited subsample, these are considered preliminary findings that should be examined in a fully powered study.

### Associations Between Inhibitory Control and Dietary Constructs

In the small subsample of participants who completed the NIH Toolbox flanker task to measure inhibitory control (19/43, 44%), inhibitory control was significantly and positively associated with sugar intake as measured through the laboratory-based administration of the dietary recall (*F*_10_=18.5; *P*=.006), with an effect size (η^2^) of 0.979 (95% CI 0.43-0.97). Inhibitory control was also marginally related to binge eating (*F*_13_=4.53; *P*=.05; η^2^=0.922, 95% CI 0.00-0.89) and cognitive restraint (*F*_13_=4.05; *P*=.06; η^2^=0.913, 95% CI 0.00-0.87) as measured using the EPSI. There were no significant associations or trends between inhibitory control and hedonic appetite or food consumption at the laboratory visit. It must be noted that this is a limited subsample and the associations presented do not meet conventional significance thresholds. These findings represent preliminary patterns that should be investigated in fully powered studies.

## Discussion

### Principal Findings

This study found that the food-specific GNG ICT intervention had preliminary associations with attentional bias toward food cues as measured through an eye tracking gaze fixation task compared to a control GNG ICT task. Additionally, inhibitory control as measured using the NIH Toolbox was significantly associated with sugar intake as measured using the dietary recall completed at the laboratory visit and showed marginally significant cross-sectional associations with binge eating and cognitive restraint. Our findings contribute to the literature examining the mechanisms through which ICT functions. The understanding of these mechanisms is still evolving within the field, with a few possible pathways, including the devaluation processes [47]. This means that attention is often allocated toward rewarding palatable food stimuli; therefore, decreasing the reward value of palatable food stimuli is a *possible* mechanism that could be at the core of these processes. Still, attentional bias has had mixed associations with palatable food intake thus far [5,48,49]. Further research is needed to examine the role of attention allocation in predicting eating behavior in adolescents and whether ICT is the most effective intervention.

Contrary to the hypotheses, the GNG intervention did not have any effect on hedonic appetite or food consumption measured either in the laboratory or via the dietary recall, and inhibitory control was not related to hedonic appetite or food consumption in the laboratory visit. While this study was indeed limited by the small sample size, we note that there are some mixed associations between inhibitory control and eating behavior in adolescents in the literature, with some studies showing no association [50] and others showing low inhibitory control as a risk factor for developing disordered eating [51]. While a large portion of the literature has focused on inhibitory control–binge eating relationships in the context of obesity, inhibitory control difficulties have been linked to loss-of-control eating in community samples of adolescents as well [2]. Interestingly, in our sample, inhibitory control was related to sugar consumption and binge eating in the opposite direction than hypothesized. In this small sample, higher inhibitory control was associated with higher sugar consumption and risk of binge eating. Considering this unexpected association and the fact that inhibitory control was also marginally associated with cognitive restraint, the mechanistic role of inhibitory control should be examined in fully powered studies aligned with the neurobehavioral inhibitory control model [7].

### Study Strengths and Limitations

This study’s strengths lie in the use of objective and gold-standard measurements, including the eye tracking gaze fixation task for attentional bias toward food cues, dietary recalls, objective measurement of palatable food consumption in the laboratory visit “taste test,” and the NIH Toolbox, methods that could all be used in a future fully powered study. A main limitation of this study is the small sample size, which was an unanticipated change due to the COVID-19 pandemic that resulted in discontinuing data collection in March 2020. This study is also limited by administration of the eye tracking task and the NIH Toolbox in subsamples of our total sample. This was done to pilot each methodology with the adolescent age group and as part of the laboratory visit procedures overall. Because only a subsample completed the eye tracking and NIH Toolbox tasks, we cannot make any claims about external validity. It must be noted that these small subsamples limit power and generalizability and cannot be taken as definitive. The findings should be considered preliminary, and these procedures should be conducted in fully powered studies as a next step. Additionally, participants in this study were predominantly White, female, and from higher-income households. Recruiting more demographically diverse individuals is a necessary focus for future research in this area, especially as systemic inequities based on race and ethnicity, socioeconomic status (eg, food insecurity), and cultural factors influence eating behavior and overall health [52]. The lack of diversity in this study also limits the applicability of the results to broader adolescent populations, which can be improved upon in larger, fully powered, and representative studies.

### Conclusions and Future Directions

Considering the need for real-time and objective measurement of appetite and eating, studies in which the data capture, intervention, and assessment occur in real time may be better suited for continued investigations. Moreover, theoretical models of dietary behavior can potentially be enhanced by expanding to include dynamic processes. For example, dynamically assessed motivational processes and behavioral outputs of the neurobehavioral inhibitory control model of feeding, such as inhibitory control, hedonic appetite, delay discounting, selective attention, and feeding behavior [3], could be included in a model as there is growing evidence that all of them can be measured successfully and show significant associations when measured via ecological momentary assessment (EMA). Knowing that individual differences (eg, motivation, weight status, and intentions to restrict intake) affect both dietary behavior [53] and the outcome of GNG interventions [54], capturing these processes more fully via EMA appears to be a promising avenue [55]. Studies focused on the adolescent age group specifically should also include adolescent-specific factors that impact dietary behavior, such as the social influences of peer groups and cognitive factors in this developmental period of increasing autonomy [53]. In conjunction with this, some studies have used internet platforms [34], video games [56], and virtual reality [26] as methods of administering GNG interventions, which may be promising for future EMA protocols especially targeting adolescents. Although our larger study included evening surveys to examine within-person processes [31], it was still not fully powered to examine the effects of the GNG intervention on fluctuating mechanisms. Rigorous and fully powered study designs that capture additional drivers of dietary behavior in real time as they fluctuate, such as access to food across environments, social cues, and attention toward food cues [3], will also improve the potential impact and application of the findings. Additionally, in this study, we piloted a single-session GNG ICT intervention, whereas other potential avenues for this research could include a longer duration or multiple sessions, alternate methods of capturing state change, and other conceptualizations of how to intervene on hedonic appetite examined longitudinally.

The role of inhibitory control in models of dysregulated eating and weight-related health continues to be a focus of investigation [13]. Although no claims about clinical utility can be made from our pilot study, recent work from other teams can inform next steps for research in this area. For example, Manasse et al [26] have found preliminary effects of a daily administered virtual reality ICT intervention on loss-of-control eating. Leaders in this literature acknowledge that clinical interventions for dysregulated eating conditions often result in limited improvement due to inadequately addressing inhibitory control and factors driving binge eating [57]. Given its evidence of reducing binge eating [58], continued application of ICT is warranted to determine effectiveness and understand mechanisms, but additional intervention avenues must also be explored. A recent systematic review of inhibitory control, loss-of-control eating, and reward sensitivity in children and adolescents also called for more research examining cognitive mechanisms longitudinally in youth, especially through use of EMA [2]. There is potential for cognitive interventions used in the key developmental period of adolescence to prevent the maladaptive dietary processes and problematic eating behaviors that often underlie both obesity risk and eating psychopathology [59]. Additional interventions targeting intuitive eating [60] and mindful decision-making [61], improvement of state and trait mindfulness [62], and understanding of the role of affect regulation [63] to promote healthful eating behavior have also shown promise for prevention of disordered eating, and their comparative value should be examined in adolescent samples.

## Appendix

Multimedia Appendix 1 Images used in go/no go task.

Multimedia Appendix 2 CONSORT-eHEALTH checklist (V 1.6.1).

## Abbreviations

ASA24: Automated Self-Administered 24-Hour Dietary Assessment Tool
EMA: ecological momentary assessment
EPSI: Eating Pathology Symptoms Inventory
GNG: go/no go
ICT: inhibitory control training
NIH: National Institutes of Health
PFS: Power of Food Scale

## Data Availability

Deidentified data from this study are not available in a public archive and will be made available (as permissible according to institutional review board standards) upon reaching out to the corresponding author.
