# Supplementary material for: Inhibitory Control, Attentional Bias, and Palatable Food Consumption in Adolescents: A Laboratory Feasibility Randomized Controlled Trial
Source: JMIR Form Res. 2025 Dec 5;9:e77579. doi: 10.2196/77579 (PMC12717506; doi:10.2196/77579)
Supplement: Multimedia Appendix 1 [file formative_v9i1e77579_app1.docx]

**Appendix A: *Food-pics* images**

Images used for the food and animal stimuli in the GNG tasks were used with formal approval from a food-pics image database designed to facilitate standardization and comparability across studies of eating behavior:

Blechert, J., Meule, A., Busch, N. A., & Ohla, K. (2014). Food-pics: an image database for

experimental research on eating and appetite. *Frontiers in Psychology, 5*, 617. <https://doi.org/10.3389/fpsyg.2014.00617>

The following specific *food-pics* image numbers were used in this study. Images of gummy bears (0157) and horse (1167) appear in Figure 1.

0026 potato chips

0046 fried

0053 hotdog

0061 pizza

0065 burger

0073 pasta

0157 gummy bears

0169 donut

0296 M&Ms

0298 cookie

0300 bread

0372 cereal

0488 waffle

0563 steak

1160 rhino

1161 fish1

1162 fish2

1167 horse

1171 elephant

1177 chick

1181 dog1

1179 dog2

1180 dog3

1183 fox

1184 polar bear

1185 cat

1186 dog4

1189 penguin

1192 frog

1193 bird
