# Supplementary material for: Inhibitory Control, Attentional Bias, and Palatable Food Consumption in Adolescents: A Laboratory Feasibility Randomized Controlled Trial
Source: JMIR Form Res. 2025 Dec 5;9:e77579. doi: 10.2196/77579 (PMC12717506; doi:10.2196/77579)
Supplement: Multimedia Appendix 2 [file formative_v9i1e77579_app2.pdf]

CONSORT-EHEALTH (V 1.6.1) - Submission/Publication Form

The CONSORT-EHEALTH checklist is intended for authors of randomized trials evaluating web-based and Internet-based applications/interventions, including mobile interventions, electronic games (incl multiplayer games), social media, certain telehealth applications, and other interactive and/or networked electronic applications. Some of the items (e.g. all subitems under item 5 - description of the intervention) may also be applicable for other study designs.

The goal of the CONSORT EHEALTH checklist and guideline is to be
a) a guide for reporting for authors of RCTs,
b) to form a basis for appraisal of an ehealth trial (in terms of validity)

CONSORT-EHEALTH items/subitems are MANDATORY reporting items for studies published in the Journal of Medical Internet Research and other journals / scientific societies endorsing the checklist.

Items numbered 1., 2., 3., 4a., 4b etc are original CONSORT or CONSORT-NPT (non-pharmacologic treatment) items.
Items with Roman numerals (i., ii, iii, iv etc.) are CONSORT-EHEALTH extensions/clarifications.

As the CONSORT-EHEALTH checklist is still considered in a formative stage, we would ask that you also RATE ON A SCALE OF 1-5 how important/useful you feel each item is FOR THE PURPOSE OF THE CHECKLIST and reporting guideline (optional).

Mandatory reporting items are marked with a red *.
In the textboxes, either copy & paste the relevant sections from your manuscript into this form - please include any quotes from your manuscript in QUOTATION MARKS,
or answer directly by providing additional information not in the manuscript, or elaborating on why the item was not relevant for this study.

YOUR ANSWERS WILL BE PUBLISHED AS A SUPPLEMENTARY FILE TO YOUR PUBLICATION IN JMIR AND ARE CONSIDERED PART OF YOUR PUBLICATION (IF ACCEPTED).
Please fill in these questions diligently. Information will not be copyedited, so please use proper spelling and grammar, use correct capitalization, and avoid abbreviations.

DO NOT FORGET TO SAVE AS PDF _AND_ CLICK THE SUBMIT BUTTON SO YOUR ANSWERS ARE IN OUR DATABASE !!!

Citation Suggestion (if you append the pdf as Appendix we suggest to cite this paper in the caption):
Eysenbach G, CONSORT-EHEALTH Group

CONSORT-EHEALTH: Improving and Standardizing Evaluation Reports of Web-based and Mobile Health Interventions
J Med Internet Res 2011;13(4):e126
URL: http://www.jmir.org/2011/4/e126/
doi: 10.2196/jmir.1923
PMID: 22209829

carolina11bejarano@gmail.com Switch account

Not shared

Draft saved

* Indicates required question

Your name *

First Last

Carolina Bejarano

Primary Affiliation (short), City, Country *

University of Toronto, Toronto, Canada

Cincinnati Children's Hospital Medical Center; [illegible]

Your e-mail address *

abc@gmail.com

carolina.bejarano@cchmc.org

Title of your manuscript *

Provide the (draft) title of your manuscript.

Inhibitory Control, Attentional Bias, and Palatable Food Consumption in Adolescents: A Feasibility Randomized Controlled Trial

Name of your App/Software/Intervention *

If there is a short and a long/alternate name, write the short name first and add the long name in brackets.

Food Go/No Go Inhibitory Control Training Inte

Evaluated Version (if any)

e.g. "V1", "Release 2017-03-01", "Version 2.0.27913"

Your answer

Language(s) *

What language is the intervention/app in? If multiple languages are available, separate by comma (e.g. "English, French")

English

URL of your Intervention Website or App

e.g. a direct link to the mobile app on app in appstore (itunes, Google Play), or URL of the website. If the intervention is a DVD or hardware, you can also link to an Amazon page.

Your answer

URL of an image/screenshot (optional)

Your answer

Accessibility *

Can an enduser access the intervention presently?

- ( ) access is free and open
- ( ) access only for special usergroups, not open
- ( ) access is open to everyone, but requires payment/subscription/in-app purchases
- (•) app/intervention no longer accessible
- ( ) Other:

Primary Medical Indication/Disease/Condition *

e.g. "Stress", "Diabetes", or define the target group in brackets after the condition, e.g. "Autism (Parents of children with)", "Alzheimers (Informal Caregivers of)"

Adolescent dietary behavior

Primary Outcomes measured in trial *

comma-separated list of primary outcomes reported in the trial

attentional bias to food cues, hedonic appetite

Secondary/other outcomes

Are there any other outcomes the intervention is expected to affect?

Your answer

Recommended "Dose" *

What do the instructions for users say on how often the app should be used?

- ( ) Approximately Daily
- ( ) Approximately Weekly
- ( ) Approximately Monthly
- ( ) Approximately Yearly
- ( ) "as needed"
- (x) Other: to be determined, these are preliminary results of a feasibility trial

Approx. Percentage of Users (starters) still using the app as recommended after 3 months *

- (x) unknown / not evaluated
- ( ) 0-10%
- ( ) 11-20%
- ( ) 21-30%
- ( ) 31-40%
- ( ) 41-50%
- ( ) 51-60%
- ( ) 61-70%
- ( ) 71%-80%
- ( ) 81-90%
- ( ) 91-100%
- ( ) Other:

Overall, was the app/intervention effective? *

- ( ) yes: all primary outcomes were significantly better in intervention group vs control
- (x) partly: SOME primary outcomes were significantly better in intervention group vs control
- ( ) no statistically significant difference between control and intervention
- ( ) potentially harmful: control was significantly better than intervention in one or more outcomes
- ( ) inconclusive: more research is needed
- ( ) Other:

Article Preparation Status/Stage *

At which stage in your article preparation are you currently (at the time you fill in this form)

- ( ) not submitted yet - in early draft status
- ( ) not submitted yet - in late draft status, just before submission
- ( ) submitted to a journal but not reviewed yet
- ( ) submitted to a journal and after receiving initial reviewer comments
- (x) submitted to a journal and accepted, but not published yet
- ( ) published
- ( ) Other:

Journal *

If you already know where you will submit this paper (or if it is already submitted), please provide the journal name (if it is not JMIR, provide the journal name under "other")

- ○ not submitted yet / unclear where I will submit this
- ○ Journal of Medical Internet Research (JMIR)
- ○ JMIR mHealth and UHealth
- ○ JMIR Serious Games
- ○ JMIR Mental Health
- ○ JMIR Public Health
- ◉ JMIR Formative Research
- ○ Other JMIR sister journal
- ○ Other:

Is this a full powered effectiveness trial or a pilot/feasibility trial? *

- ◉ Pilot/feasibility
- ○ Fully powered

Manuscript tracking number *

If this is a JMIR submission, please provide the manuscript tracking number under "other" (The ms tracking number can be found in the submission acknowledgement email, or when you login as author in JMIR. If the paper is already published in JMIR, then the ms tracking number is the four-digit number at the end of the DOI, to be found at the bottom of each published article in JMIR)

- ○ no ms number (yet) / not (yet) submitted to / published in JMIR
- ◉ Other: 77579

TITLE AND ABSTRACT

1a) TITLE: Identification as a randomized trial in the title

1a) Does your paper address CONSORT item 1a? *

I.e does the title contain the phrase "Randomized Controlled Trial"? (if not, explain the reason under "other")

- (x) yes
- ( ) Other:

1a-i) Identify the mode of delivery in the title

Identify the mode of delivery. Preferably use “web-based” and/or “mobile” and/or “electronic game” in the title. Avoid ambiguous terms like “online”, “virtual”, “interactive”. Use “Internet-based” only if Intervention includes non-web-based Internet components (e.g. email), use “computer-based” or “electronic” only if offline products are used. Use “virtual” only in the context of “virtual reality” (3-D worlds). Use “online” only in the context of “online support groups”. Complement or substitute product names with broader terms for the class of products (such as “mobile” or “smart phone” instead of “iphone”), especially if the application runs on different platforms.

	1	2	3	4	5	
subitem not at all important	( )	( )	( )	( )	( )	essential

Does your paper address subitem 1a-i? *

Copy and paste relevant sections from manuscript title (include quotes in quotation marks "like this" to indicate direct quotes from your manuscript), or elaborate on this item by providing additional information not in the ms, or briefly explain why the item is not applicable/relevant for your study

Yes, this was an intervention conducted on a computer

1a-ii) Non-web-based components or important co-interventions in title

Mention non-web-based components or important co-interventions in title, if any (e.g., “with telephone support”).

	1	2	3	4	5	
subitem not at all important	○	○	○	○	○	essential

Does your paper address subitem 1a-ii?

Copy and paste relevant sections from manuscript title (include quotes in quotation marks "like this" to indicate direct quotes from your manuscript), or elaborate on this item by providing additional information not in the ms, or briefly explain why the item is not applicable/relevant for your study

Your answer

1a-iii) Primary condition or target group in the title

Mention primary condition or target group in the title, if any (e.g., “for children with Type I Diabetes”) Example: A Web-based and Mobile Intervention with Telephone Support for Children with Type I Diabetes: Randomized Controlled Trial

	1	2	3	4	5	
subitem not at all important	○	○	○	○	○	essential

Does your paper address subitem 1a-iii? *

Copy and paste relevant sections from manuscript title (include quotes in quotation marks "like this" to indicate direct quotes from your manuscript), or elaborate on this item by providing additional information not in the ms, or briefly explain why the item is not applicable/relevant for your study

Yes, this was a study of adolescents

1b) ABSTRACT: Structured summary of trial design, methods, results, and conclusions

NPT extension: Description of experimental treatment, comparator, care providers, centers, and blinding status.

1b-i) Key features/functionalities/components of the intervention and comparator in the METHODS section of the ABSTRACT

Mention key features/functionalities/components of the intervention and comparator in the abstract. If possible, also mention theories and principles used for designing the site. Keep in mind the needs of systematic reviewers and indexers by including important synonyms. (Note: Only report in the abstract what the main paper is reporting. If this information is missing from the main body of text, consider adding it)

	1	2	3	4	5	
subitem not at all important	○	○	○	○	○	essential

Does your paper address subitem 1b-i? *

Copy and paste relevant sections from the manuscript abstract (include quotes in quotation marks "like this" to indicate direct quotes from your manuscript), or elaborate on this item by providing additional information not in the ms, or briefly explain why the item is not applicable/relevant for your study

Yes, participants were randomized to a food-cue specific Go/No Go inhibitory control training task or a control (non food-cue specific) Go/No Go Inhibitory control training task.

1b-ii) Level of human involvement in the METHODS section of the ABSTRACT

Clarify the level of human involvement in the abstract, e.g., use phrases like “fully automated” vs. “therapist/nurse/care provider/physician-assisted” (mention number and expertise of providers involved, if any). (Note: Only report in the abstract what the main paper is reporting. If this information is missing from the main body of text, consider adding it)

	1	2	3	4	5	
subitem not at all important	○	○	○	○	○	essential

Does your paper address subitem 1b-ii?

Copy and paste relevant sections from the manuscript abstract (include quotes in quotation marks "like this" to indicate direct quotes from your manuscript), or elaborate on this item by providing additional information not in the ms, or briefly explain why the item is not applicable/relevant for your study

Your answer

1b-iii) Open vs. closed, web-based (self-assessment) vs. face-to-face assessments in the METHODS section of the ABSTRACT

Mention how participants were recruited (online vs. offline), e.g., from an open access website or from a clinic or a closed online user group (closed usergroup trial), and clarify if this was a purely web-based trial, or there were face-to-face components (as part of the intervention or for assessment). Clearly say if outcomes were self-assessed through questionnaires (as common in web-based trials). Note: In traditional offline trials, an open trial (open-label trial) is a type of clinical trial in which both the researchers and participants know which treatment is being administered. To avoid confusion, use “blinded” or “unblinded” to indicated the level of blinding instead of “open”, as “open” in web-based trials usually refers to “open access” (i.e. participants can self-enrol). (Note: Only report in the abstract what the main paper is reporting. If this information is missing from the main body of text, consider adding it)

	1	2	3	4	5	
subitem not at all important	○	○	○	○	○	essential

Does your paper address subitem 1b-iii?

Copy and paste relevant sections from the manuscript abstract (include quotes in quotation marks "like this" to indicate direct quotes from your manuscript), or elaborate on this item by providing additional information not in the ms, or briefly explain why the item is not applicable/relevant for your study

Your answer

1b-iv) RESULTS section in abstract must contain use data

Report number of participants enrolled/assessed in each group, the use/uptake of the intervention (e.g., attrition/adherence metrics, use over time, number of logins etc.), in addition to primary/secondary outcomes. (Note: Only report in the abstract what the main paper is reporting. If this information is missing from the main body of text, consider adding it)

	1	2	3	4	5	
subitem not at all important	○	○	○	○	○	essential

Does your paper address subitem 1b-iv?

Copy and paste relevant sections from the manuscript abstract (include quotes in quotation marks "like this" to indicate direct quotes from your manuscript), or elaborate on this item by providing additional information not in the ms, or briefly explain why the item is not applicable/relevant for your study

Your answer

1b-v) CONCLUSIONS/DISCUSSION in abstract for negative trials

Conclusions/Discussions in abstract for negative trials: Discuss the primary outcome - if the trial is negative (primary outcome not changed), and the intervention was not used, discuss whether negative results are attributable to lack of uptake and discuss reasons. (Note: Only report in the abstract what the main paper is reporting. If this information is missing from the main body of text, consider adding it)

	1	2	3	4	5	
subitem not at all important	○	○	○	○	○	essential

Does your paper address subitem 1b-v?

Copy and paste relevant sections from the manuscript abstract (include quotes in quotation marks "like this" to indicate direct quotes from your manuscript), or elaborate on this item by providing additional information not in the ms, or briefly explain why the item is not applicable/relevant for your study

Your answer

INTRODUCTION

2a) In INTRODUCTION: Scientific background and explanation of rationale

2a-i) Problem and the type of system/solution

Describe the problem and the type of system/solution that is object of the study: intended as stand-alone intervention vs. incorporated in broader health care program? Intended for a particular patient population? Goals of the intervention, e.g., being more cost-effective to other interventions, replace or complement other solutions? (Note: Details about the intervention are provided in “Methods” under 5)

	1	2	3	4	5	
subitem not at all important	○	○	○	○	○	essential

Does your paper address subitem 2a-i? *

Copy and paste relevant sections from the manuscript (include quotes in quotation marks "like this" to indicate direct quotes from your manuscript), or elaborate on this item by providing additional information not in the ms, or briefly explain why the item is not applicable/relevant for your study

"The literature to date indicates that the field is lacking studies that examine associations of inhibitory control in the context of dietary behavior in non-clinical samples of adolescents, through both objective laboratory-based and subjective self-report outcomes [2]. An enhanced understanding of processes impacting dietary behavior in adolescents can be harnessed to inform preventive interventions geared toward improving health and well-being in this age group as well as in the long- term into adulthood. The limited previous research to date has found that inhibitory control has been inversely related to facets of binge eating in community samples of adolescents and reward sensitivity has been related to binge eating behaviors in youth [2]. Together with the theoretical underpinnings of the neurobehavioral inhibitory control model of feeding [3] and the background literature described previously, this study was designed to examine the preliminary effects and pilot methodology of a randomized laboratory study of ICT and to examine mechanisms of inhibitory control, attentional bias to food cues, hedonic appetite, palatable food consumption, and binge eating in a non-clinical sample of adolescents."

2a-ii) Scientific background, rationale: What is known about the (type of) system

Scientific background, rationale: What is known about the (type of) system that is the object of the study (be sure to discuss the use of similar systems for other conditions/diagnoses, if appropiate), motivation for the study, i.e. what are the reasons for and what is the context for this specific study, from which stakeholder viewpoint is the study performed, potential impact of findings [2]. Briefly justify the choice of the comparator.

	1	2	3	4	5	
subitem not at all important	○	○	○	○	○	essential

Does your paper address subitem 2a-ii? *

Copy and paste relevant sections from the manuscript (include quotes in quotation marks "like this" to indicate direct quotes from your manuscript), or elaborate on this item by providing additional information not in the ms, or briefly explain why the item is not applicable/relevant for your study

"Evidence-based methods used to study cognitive processes in the context of dietary behavior include reaction time paradigms to assess and intervene on inhibitory control [20] and eye tracking tasks to measure attentional bias toward food cues [21]. Food-specific inhibitory control training (ICT) tasks have been used to influence food choices [22]. A go/no go (GNG) ICT task is a procedure in which 2 categories of visual stimuli are randomly presented and a respondent is required to respond to images from one stimuli category (go) while withholding a response to an alternative stimuli category (no go) [23]. There is a majority of evidence suggesting that GNG training creates automatic stop associations that can influence outcomes of food choice, portion size, and weight changes [24] by training an individual to inhibit a response to food cues [22]. The few studies on GNG training in youth have found relationships with adolescents' inhibitory control for intake of sugar-sweetened beverages [12] and reduction in consumption of energy-dense candy in children when measured in laboratory settings [25]. One pilot study found that a virtual reality ICT resulted in reduced binge eating up to the 2-week follow-up in adults [26]. While a GNG ICT task is often used as an intervention, a flanker task is an executive function measure that is used as an assessment of inhibitory control. The flanker task assesses a participant's ability to allocate cognitive resources to process stimuli while also ignoring extraneous stimuli [27]. Finally, gaze fixation tasks with an eye tracker are often used to measure attentional bias toward food cues by measuring overall gaze direction and duration bias [28]. In adolescents, studies using gaze fixation with eye tracking data have found greater gaze duration bias toward food cues in a group with binge eating as compared to the control group [29]."

2b) In INTRODUCTION: Specific objectives or hypotheses

Does your paper address CONSORT subitem 2b? *

Copy and paste relevant sections from the manuscript (include quotes in quotation marks "like this" to indicate direct quotes from your manuscript), or elaborate on this item by providing additional information not in the ms, or briefly explain why the item is not applicable/relevant for your study

"The following hypotheses were tested: (1) the food-specific GNG task will show preliminary associations with reductions in attention to palatable food cues, hedonic appetite, palatable food consumption in a laboratory-based eating paradigm, and food intake via dietary recall; and (2) inhibitory control will show preliminary inverse associations with hedonic appetite, palatable food consumption (in a laboratory-based paradigm and via dietary recall), and binge eating and preliminary positive associations with cognitive restraint."

METHODS

3a) Description of trial design (such as parallel, factorial) including allocation ratio

Does your paper address CONSORT subitem 3a? *

Copy and paste relevant sections from the manuscript (include quotes in quotation marks "like this" to indicate direct quotes from your manuscript), or elaborate on this item by providing additional information not in the ms, or briefly explain why the item is not applicable/relevant for your study

Pilot and feasibility randomized controlled trial

3b) Important changes to methods after trial commencement (such as eligibility criteria), with reasons

Does your paper address CONSORT subitem 3b? *

Copy and paste relevant sections from the manuscript (include quotes in quotation marks "like this" to indicate direct quotes from your manuscript), or elaborate on this item by providing additional information not in the ms, or briefly explain why the item is not applicable/relevant for your study

Not applicable, there were no changes to methods after trail commencement

3b-i) Bug fixes, Downtimes, Content Changes

Bug fixes, Downtimes, Content Changes: ehealth systems are often dynamic systems. A description of changes to methods therefore also includes important changes made on the intervention or comparator during the trial (e.g., major bug fixes or changes in the functionality or content) (5-iii) and other "unexpected events" that may have influenced study design such as staff changes, system failures/downtimes, etc. [2].

	1	2	3	4	5	
subitem not at all important	○	○	○	○	○	essential

Does your paper address subitem 3b-i?

Copy and paste relevant sections from the manuscript (include quotes in quotation marks "like this" to indicate direct quotes from your manuscript), or elaborate on this item by providing additional information not in the ms, or briefly explain why the item is not applicable/relevant for your study

Your answer

4a) Eligibility criteria for participants

Does your paper address CONSORT subitem 4a? *

Copy and paste relevant sections from the manuscript (include quotes in quotation marks "like this" to indicate direct quotes from your manuscript), or elaborate on this item by providing additional information not in the ms, or briefly explain why the item is not applicable/relevant for your study

"To best control for the food environment across participants and study dietary behavior in typically functioning adolescents, the inclusion criteria were living in a home setting with their parents or caregivers, the ability to read at their grade level and in English, and absence of blindness or low vision. The exclusion criteria were presence of developmental delays, past or current self-reported eating disorder diagnoses, and any health conditions or dietary restrictions that significantly affected activity or eating habits (e.g., celiac disease or vegan diet)."

4a-i) Computer / Internet literacy

Computer / Internet literacy is often an implicit “de facto” eligibility criterion - this should be explicitly clarified.

	1	2	3	4	5	
subitem not at all important	○	○	○	○	○	essential

Does your paper address subitem 4a-i?

Copy and paste relevant sections from the manuscript (include quotes in quotation marks "like this" to indicate direct quotes from your manuscript), or elaborate on this item by providing additional information not in the ms, or briefly explain why the item is not applicable/relevant for your study

Your answer

4a-ii) Open vs. closed, web-based vs. face-to-face assessments:

Open vs. closed, web-based vs. face-to-face assessments: Mention how participants were recruited (online vs. offline), e.g., from an open access website or from a clinic, and clarify if this was a purely web-based trial, or there were face-to-face components (as part of the intervention or for assessment), i.e., to what degree got the study team to know the participant. In online-only trials, clarify if participants were quasi-anonymous and whether having multiple identities was possible or whether technical or logistical measures (e.g., cookies, email confirmation, phone calls) were used to detect/prevent these.

	1	2	3	4	5	
subitem not at all important	○	○	○	○	○	essential

Does your paper address subitem 4a-ii? *

Copy and paste relevant sections from the manuscript (include quotes in quotation marks "like this" to indicate direct quotes from your manuscript), or elaborate on this item by providing additional information not in the ms, or briefly explain why the item is not applicable/relevant for your study

"A total of 43 adolescent participants aged 13 to 18 years were recruited through the local community for a study about adolescent health behaviors."

4a-iii) Information giving during recruitment

Information given during recruitment. Specify how participants were briefed for recruitment and in the informed consent procedures (e.g., publish the informed consent documentation as appendix, see also item X26), as this information may have an effect on user self-selection, user expectation and may also bias results.

	1	2	3	4	5	
subitem not at all important	○	○	○	○	○	essential

Does your paper address subitem 4a-iii?

Copy and paste relevant sections from the manuscript (include quotes in quotation marks "like this" to indicate direct quotes from your manuscript), or elaborate on this item by providing additional information not in the ms, or briefly explain why the item is not applicable/relevant for your study

Your answer

4b) Settings and locations where the data were collected

Does your paper address CONSORT subitem 4b? *

Copy and paste relevant sections from the manuscript (include quotes in quotation marks "like this" to indicate direct quotes from your manuscript), or elaborate on this item by providing additional information not in the ms, or briefly explain why the item is not applicable/relevant for your study

The study was conducted and data was collected in person at the laboratory visit.

4b-i) Report if outcomes were (self-)assessed through online questionnaires

Clearly report if outcomes were (self-)assessed through online questionnaires (as common in web-based trials) or otherwise.

	1	2	3	4	5	
subitem not at all important	○	○	○	○	○	essential

Does your paper address subitem 4b-i? *

Copy and paste relevant sections from the manuscript (include quotes in quotation marks "like this" to indicate direct quotes from your manuscript), or elaborate on this item by providing additional information not in the ms, or briefly explain why the item is not applicable/relevant for your study

Some outcomes were objectively measured and some were assessed through online questionnaires:

"Bogus Taste Test

After completing the GNG training, participants completed a hedonic appetite survey measure and then were brought to a separate room with a comfortable chair, an iPad playing a television show, and large amounts of the preferred palatable foods. Participants were allowed 20 minutes to complete the taste test and instructed to eat as much as they liked. The amount of each type of food eaten by each participant was weighed covertly by the masked research assistant to calculate the amount of palatable food consumed.

Eye Tracker and Remaining Tasks

A gaze fixation task with the use of an eye tracker was administered on a desktop computer to a subset of the study participants to determine attentional bias for food versus nonfood images. Participants then each completed self-report questionnaires, a dietary recall, and cognitive tasks and had their height and weight recorded. After the last day of study procedures, participants and parents were mailed their gift card compensation along with a debriefing letter outlining the research questions of interest.

Constructs and Measures

Demographics and Anthropometrics

Participants completed a demographic questionnaire at the laboratory visit. Each participant’s height and weight were measured at the end of the laboratory visit following standard protocols. BMI percentile was calculated based on age and sex as indicated by the Centers for Disease Control and Prevention [36].

Food Preferences

The Food Preference Questionnaire was designed specifically for this study based on factor loadings from the Food Craving Inventory (ie, sweet, starchy, fatty, and fast food [30]) and on studies examining consumption of palatable food in a laboratory setting [7]. In this sample, the Food Craving Inventory demonstrated good internal consistency reliability (Cronbach α=0.93 for the total, 0.86 for sweet, 0.84 for starchy, 0.86 for fatty, and 0.76 for fast food). Participants completed this before the laboratory visit to determine which foods would be available during their bogus taste test.

Hedonic Appetite

The Power of Food Scale (PFS) is a 15-item measure that assesses the construct of hedonic appetite [37,38] using a 5-point Likert scale. Higher mean scores indicate higher hedonic appetite (range 1-5). The PFS has shown high internal consistency reliability in a previous sample of healthy adolescents (Cronbach α=0.94 [31]). The PFS was completed after completion of the GNG task.

Palatable Food Consumption

The amounts of the 4 foods offered in the bogus taste test were standardized based on caloric content. More specifically, research staff were trained to reliably measure and serve 500 to 600 calories of each food offered in the study. After the bogus taste test, the remaining portions of each food were weighed covertly on a food scale in grams, and the amount consumed was converted to calories.

Typical Dietary Intake

The ASA24 is a freely available web-based tool developed by the US National Cancer Institute [39] to obtain high-quality daily dietary data with minimal bias [40]. The 1-day dietary recall is considered acceptable as a valid assessment of typical dietary intake [40]. Two administrations of the ASA24 dietary recall took place: one at the laboratory visit and one 3 days after the laboratory visit.

[illegible]

Attentional Bias to Food Cues
Attentional bias was measured through administration of the gaze fixation task with the use of an eye tracker set up on a desktop computer. The amount of time participants spent looking at food versus nonfood images (referred to as dwell time bias or gaze duration bias) was used as a measure of attentional bias to food cues in the GNG intervention group as compared to the GNG control group. This measure of gaze fixation as attentional bias has shown excellent internal reliability and acceptable test-retest reliability in eye tracking procedures of this nature [41]. The eye tracking task was administered near the end of the laboratory visit procedures (ie, after the GNG task, bogus taste test, and laboratory visit surveys).
Eating Pathology
The binge eating and cognitive restraint subscales of the Eating Pathology Symptoms Inventory (EPSI) were used to measure disordered eating constructs of interest for this study. The EPSI is a well-validated 45-item self-report measure with 8 total subscales that has participants report on eating pathology in the previous 4 weeks [42] on a 5-point Likert scale. The binge eating subscale consists of 8 items, and scores range from 4 to 40. The cognitive restraint subscale measures cognitive efforts to limit or avoid eating and consists of 3 items, and scores range from 3 to 15. The binge eating subscale has shown excellent internal consistency in the sample of adolescents in this study [43], and the cognitive restraint subscale has shown good internal consistency reliability in a sample of adolescents previously [44].
Inhibitory Control
The NIH Toolbox iPad app provides access to the NIH Toolbox for Assessment of Neurological and Behavioral Function, a standard set of valid, reliable, and royalty-free tools for assessing cognitive, emotional, motor, and sensory function in participants aged 3 to 85 years. For this study, we used the NIH Toolbox Cognition: Executive Function model for assessing inhibitory control [45]. Participants completed a flanker task (Flanker Inhibitory Control and Attention Test for ages of ≥12 years; version 2.0) using the NIH Toolbox app on the iPad provided by the laboratory, which takes approximately 3 minutes to administer. The task has participants indicate the direction of an arrow that is flanked by 2 other arrows that point in either congruent or incongruent directions. The incongruent trials provide a measure of inhibitory control based on accuracy and speed, which is automatically calculated by the NIH program software. This task has shown to be a valid measure of inhibitory control and attention in youth [46]."

4b-ii) Report how institutional affiliations are displayed

Report how institutional affiliations are displayed to potential participants [on ehealth media], as affiliations with prestigious hospitals or universities may affect volunteer rates, use, and reactions with regards to an intervention.(Not a required item – describe only if this may bias results)

	1	2	3	4	5	
subitem not at all important	○	○	○	○	○	essential

Does your paper address subitem 4b-ii?

Copy and paste relevant sections from the manuscript (include quotes in quotation marks "like this" to indicate direct quotes from your manuscript), or elaborate on this item by providing additional information not in the ms, or briefly explain why the item is not applicable/relevant for your study

Your answer

5) The interventions for each group with sufficient details to allow replication, including how and when they were actually administered

5-i) Mention names, credential, affiliations of the developers, sponsors, and owners

Mention names, credential, affiliations of the developers, sponsors, and owners [6] (if authors/evaluators are owners or developer of the software, this needs to be declared in a “Conflict of interest” section or mentioned elsewhere in the manuscript).

	1	2	3	4	5	
subitem not at all important	○	○	○	○	○	essential

Does your paper address subitem 5-i?

Copy and paste relevant sections from the manuscript (include quotes in quotation marks "like this" to indicate direct quotes from your manuscript), or elaborate on this item by providing additional information not in the ms, or briefly explain why the item is not applicable/relevant for your study

Your answer

5-ii) Describe the history/development process

Describe the history/development process of the application and previous formative evaluations (e.g., focus groups, usability testing), as these will have an impact on adoption/use rates and help with interpreting results.

	1	2	3	4	5	
subitem not at all important	○	○	○	○	○	essential

Does your paper address subitem 5-ii?

Copy and paste relevant sections from the manuscript (include quotes in quotation marks "like this" to indicate direct quotes from your manuscript), or elaborate on this item by providing additional information not in the ms, or briefly explain why the item is not applicable/relevant for your study

Your answer

5-iii) Revisions and updating

Revisions and updating. Clearly mention the date and/or version number of the application/intervention (and comparator, if applicable) evaluated, or describe whether the intervention underwent major changes during the evaluation process, or whether the development and/or content was “frozen” during the trial. Describe dynamic components such as news feeds or changing content which may have an impact on the replicability of the intervention (for unexpected events see item 3b).

	1	2	3	4	5	
subitem not at all important	○	○	○	○	○	essential

Does your paper address subitem 5-iii?

Copy and paste relevant sections from the manuscript (include quotes in quotation marks "like this" to indicate direct quotes from your manuscript), or elaborate on this item by providing additional information not in the ms, or briefly explain why the item is not applicable/relevant for your study

Your answer

5-iv) Quality assurance methods

Provide information on quality assurance methods to ensure accuracy and quality of information provided [1], if applicable.

	1	2	3	4	5	
subitem not at all important	○	○	○	○	○	essential

Does your paper address subitem 5-iv?

Copy and paste relevant sections from the manuscript (include quotes in quotation marks "like this" to indicate direct quotes from your manuscript), or elaborate on this item by providing additional information not in the ms, or briefly explain why the item is not applicable/relevant for your study

Your answer

5-v) Ensure replicability by publishing the source code, and/or providing screenshots/screen-capture video, and/or providing flowcharts of the algorithms used

Ensure replicability by publishing the source code, and/or providing screenshots/screen-capture video, and/or providing flowcharts of the algorithms used. Replicability (i.e., other researchers should in principle be able to replicate the study) is a hallmark of scientific reporting.

	1	2	3	4	5	
subitem not at all important	○	○	○	○	○	essential

Does your paper address subitem 5-v?

Copy and paste relevant sections from the manuscript (include quotes in quotation marks "like this" to indicate direct quotes from your manuscript), or elaborate on this item by providing additional information not in the ms, or briefly explain why the item is not applicable/relevant for your study

Your answer

5-vi) Digital preservation

Digital preservation: Provide the URL of the application, but as the intervention is likely to change or disappear over the course of the years; also make sure the intervention is archived (Internet Archive, webcitation.org, and/or publishing the source code or screenshots/videos alongside the article). As pages behind login screens cannot be archived, consider creating demo pages which are accessible without login.

	1	2	3	4	5	
subitem not at all important	○	○	○	○	○	essential

Does your paper address subitem 5-vi?

Copy and paste relevant sections from the manuscript (include quotes in quotation marks "like this" to indicate direct quotes from your manuscript), or elaborate on this item by providing additional information not in the ms, or briefly explain why the item is not applicable/relevant for your study

Your answer

5-vii) Access

Access: Describe how participants accessed the application, in what setting/context, if they had to pay (or were paid) or not, whether they had to be a member of specific group. If known, describe how participants obtained “access to the platform and Internet” [1]. To ensure access for editors/reviewers/readers, consider to provide a “backdoor” login account or demo mode for reviewers/readers to explore the application (also important for archiving purposes, see vi).

	1	2	3	4	5	
subitem not at all important	○	○	○	○	○	essential

Does your paper address subitem 5-vii? *

Copy and paste relevant sections from the manuscript (include quotes in quotation marks "like this" to indicate direct quotes from your manuscript), or elaborate on this item by providing additional information not in the ms, or briefly explain why the item is not applicable/relevant for your study

The research coordinator set up the participant for each computer task. They did not have to pay but were compensated: "Participants and caregivers were informed that participation was voluntary and they could discontinue at any time and were offered up to US $40 in compensation and snacks."

5-viii) Mode of delivery, features/functionalities/components of the intervention and comparator, and the theoretical framework

Describe mode of delivery, features/functionalities/components of the intervention and comparator, and the theoretical framework [6] used to design them (instructional strategy [1], behaviour change techniques, persuasive features, etc., see e.g., [7, 8] for terminology). This includes an in-depth description of the content (including where it is coming from and who developed it) [1],” whether [and how] it is tailored to individual circumstances and allows users to track their progress and receive feedback” [6]. This also includes a description of communication delivery channels and – if computer-mediated communication is a component – whether communication was synchronous or asynchronous [6]. It also includes information on presentation strategies [1], including page design principles, average amount of text on pages, presence of hyperlinks to other resources, etc. [1].

	1	2	3	4	5	
subitem not at all important	○	○	○	○	○	essential

Does your paper address subitem 5-viii? *

Copy and paste relevant sections from the manuscript (include quotes in quotation marks "like this" to indicate direct quotes from your manuscript), or elaborate on this item by providing additional information not in the ms, or briefly explain why the item is not applicable/relevant for your study

"The GNG procedures were designed in accordance with previous studies [25,34], including a practice round of the task to ensure that participants could perform it correctly. The GNG task included instructions to press a button when a go cue was presented and to not press a button when a no go cue was presented. Participants in the food-specific GNG intervention condition were trained using pictures of palatable foods grouped as sweet, starchy, fatty, and fast food as no go cues, with 4 food stimuli pictures used for each category (16 in total) and 16 nonfood stimuli pictures of animals as go cues. Those in the control GNG condition completed the GNG task with all nonfood stimuli (eg, animals and shapes; Figure 1). The images in the GNG tasks were used with approval from the Food-pics image database designed to facilitate standardization and comparability across studies [35] (Multimedia Appendix 1). For both groups, each stimulus was presented for 1000 ms, and the intertrial interval was 500 ms. If the participant made an incorrect response or did not respond within 1000 ms, a red cross appeared on the screen for 500 ms (Figure 1). For both groups, 50% of the trials were go trials (ie, 16 images of animals presented 4 times each), and 50% were no go trials."

5-ix) Describe use parameters

Describe use parameters (e.g., intended “doses” and optimal timing for use). Clarify what instructions or recommendations were given to the user, e.g., regarding timing, frequency, heaviness of use, if any, or was the intervention used ad libitum.

	1	2	3	4	5	
subitem not at all important	○	○	○	○	○	essential

Does your paper address subitem 5-ix?

Copy and paste relevant sections from the manuscript (include quotes in quotation marks "like this" to indicate direct quotes from your manuscript), or elaborate on this item by providing additional information not in the ms, or briefly explain why the item is not applicable/relevant for your study

Your answer

5-x) Clarify the level of human involvement

Clarify the level of human involvement (care providers or health professionals, also technical assistance) in the e-intervention or as co-intervention (detail number and expertise of professionals involved, if any, as well as "type of assistance offered, the timing and frequency of the support, how it is initiated, and the medium by which the assistance is delivered". It may be necessary to distinguish between the level of human involvement required for the trial, and the level of human involvement required for a routine application outside of a RCT setting (discuss under item 21 – generalizability).

	1	2	3	4	5	
subitem not at all important	O	O	O	O	O	essential

Does your paper address subitem 5-x?

Copy and paste relevant sections from the manuscript (include quotes in quotation marks "like this" to indicate direct quotes from your manuscript), or elaborate on this item by providing additional information not in the ms, or briefly explain why the item is not applicable/relevant for your study

Your answer

5-xi) Report any prompts/reminders used

Report any prompts/reminders used: Clarify if there were prompts (letters, emails, phone calls, SMS) to use the application, what triggered them, frequency etc. It may be necessary to distinguish between the level of prompts/reminders required for the trial, and the level of prompts/reminders for a routine application outside of a RCT setting (discuss under item 21 – generalizability).

	1	2	3	4	5	
subitem not at all important	○	○	○	○	○	essential

Does your paper address subitem 5-xi? *

Copy and paste relevant sections from the manuscript (include quotes in quotation marks "like this" to indicate direct quotes from your manuscript), or elaborate on this item by providing additional information not in the ms, or briefly explain why the item is not applicable/relevant for your study

Not applicable, prompts were not used for the intervention.

5-xii) Describe any co-interventions (incl. training/support)

Describe any co-interventions (incl. training/support): Clearly state any interventions that are provided in addition to the targeted eHealth intervention, as ehealth intervention may not be designed as stand-alone intervention. This includes training sessions and support [1]. It may be necessary to distinguish between the level of training required for the trial, and the level of training for a routine application outside of a RCT setting (discuss under item 21 – generalizability.

	1	2	3	4	5	
subitem not at all important	○	○	○	○	○	essential

Does your paper address subitem 5-xii? *

Copy and paste relevant sections from the manuscript (include quotes in quotation marks "like this" to indicate direct quotes from your manuscript), or elaborate on this item by providing additional information not in the ms, or briefly explain why the item is not applicable/relevant for your study

Not applicable, there were no co-interventions.

6a) Completely defined pre-specified primary and secondary outcome measures, including how and when they were assessed

Does your paper address CONSORT subitem 6a? *

Copy and paste relevant sections from the manuscript (include quotes in quotation marks "like this" to indicate direct quotes from your manuscript), or elaborate on this item by providing additional information not in the ms, or briefly explain why the item is not applicable/relevant for your study

"Bogus Taste Test

After completing the GNG training, participants completed a hedonic appetite survey measure and then were brought to a separate room with a comfortable chair, an iPad playing a television show, and large amounts of the preferred palatable foods. Participants were allowed 20 minutes to complete the taste test and instructed to eat as much as they liked. The amount of each type of food eaten by each participant was weighed covertly by the masked research assistant to calculate the amount of palatable food consumed.

Eye Tracker and Remaining Tasks

A gaze fixation task with the use of an eye tracker was administered on a desktop computer to a subset of the study participants to determine attentional bias for food versus nonfood images. Participants then each completed self-report questionnaires, a dietary recall, and cognitive tasks and had their height and weight recorded. After the last day of study procedures, participants and parents were mailed their gift card compensation along with a debriefing letter outlining the research questions of interest.

Constructs and Measures

Demographics and Anthropometrics

Participants completed a demographic questionnaire at the laboratory visit. Each participant’s height and weight were measured at the end of the laboratory visit following standard protocols. BMI percentile was calculated based on age and sex as indicated by the Centers for Disease Control and Prevention [36].

Food Preferences

The Food Preference Questionnaire was designed specifically for this study based on factor loadings from the Food Craving Inventory (ie, sweet, starchy, fatty, and fast food [30]) and on studies examining consumption of palatable food in a laboratory setting [7]. In this sample, the Food Craving Inventory demonstrated good internal consistency reliability (Cronbach α=0.93 for the total, 0.86 for sweet, 0.84 for starchy, 0.86 for fatty, and 0.76 for fast food). Participants completed this before the laboratory visit to determine which foods would be available during their bogus taste test.

Hedonic Appetite

The Power of Food Scale (PFS) is a 15-item measure that assesses the construct of hedonic appetite [37,38] using a 5-point Likert scale. Higher mean scores indicate higher hedonic appetite (range 1-5). The PFS has shown high internal consistency reliability in a previous sample of healthy adolescents (Cronbach α=0.94 [31]). The PFS was completed after completion of the GNG task.

Palatable Food Consumption

The amounts of the 4 foods offered in the bogus taste test were standardized based on caloric content. More specifically, research staff were trained to reliably measure and serve 500 to 600 calories of each food offered in the study. After the bogus taste test, the remaining portions of each food were weighed covertly on a food scale in grams, and the amount consumed was converted to calories.

Typical Dietary Intake

The ASA24 is a freely available web-based tool developed by the US National Cancer Institute [39] to obtain high-quality daily dietary data with minimal bias [40]. The 1-day dietary recall is considered acceptable as a valid assessment of typical dietary intake [40]. Two administrations of the ASA24 dietary recall took place: one at the laboratory visit and one 3 days after the laboratory visit.

Attentional Bias to Food Cues

Attentional bias was measured through administration of the gaze fixation task with the use [illegible]

of an eye tracker set up on a desktop computer. The amount of time participants spent looking at food versus nonfood images (referred to as dwell time bias or gaze duration bias) was used as a measure of attentional bias to food cues in the GNG intervention group as compared to the GNG control group. This measure of gaze fixation as attentional bias has shown excellent internal reliability and acceptable test-retest reliability in eye tracking procedures of this nature [41]. The eye tracking task was administered near the end of the laboratory visit procedures (ie, after the GNG task, bogus taste test, and laboratory visit surveys).
Eating Pathology
The binge eating and cognitive restraint subscales of the Eating Pathology Symptoms Inventory (EPSI) were used to measure disordered eating constructs of interest for this study. The EPSI is a well-validated 45-item self-report measure with 8 total subscales that has participants report on eating pathology in the previous 4 weeks [42] on a 5-point Likert scale. The binge eating subscale consists of 8 items, and scores range from 4 to 40. The cognitive restraint subscale measures cognitive efforts to limit or avoid eating and consists of 3 items, and scores range from 3 to 15. The binge eating subscale has shown excellent internal consistency in the sample of adolescents in this study [43], and the cognitive restraint subscale has shown good internal consistency reliability in a sample of adolescents previously [44].
Inhibitory Control
The NIH Toolbox iPad app provides access to the NIH Toolbox for Assessment of Neurological and Behavioral Function, a standard set of valid, reliable, and royalty-free tools for assessing cognitive, emotional, motor, and sensory function in participants aged 3 to 85 years. For this study, we used the NIH Toolbox Cognition: Executive Function model for assessing inhibitory control [45]. Participants completed a flanker task (Flanker Inhibitory Control and Attention Test for ages of ≥12 years; version 2.0) using the NIH Toolbox app on the iPad provided by the laboratory, which takes approximately 3 minutes to administer. The task has participants indicate the direction of an arrow that is flanked by 2 other arrows that point in either congruent or incongruent directions. The incongruent trials provide a measure of inhibitory control based on accuracy and speed, which is automatically calculated by the NIH program software. This task has shown to be a valid measure of inhibitory control and attention in youth [46]."

6a-i) Online questionnaires: describe if they were validated for online use and apply CHERRIES items to describe how the questionnaires were designed/deployed

If outcomes were obtained through online questionnaires, describe if they were validated for online use and apply CHERRIES items to describe how the questionnaires were designed/deployed [9].

	1	2	3	4	5	
subitem not at all important	○	○	○	○	○	essential

Does your paper address subitem 6a-i?

Copy and paste relevant sections from manuscript text

Your answer

6a-ii) Describe whether and how “use” (including intensity of use/dosage) was defined/measured/monitored

Describe whether and how “use” (including intensity of use/dosage) was defined/measured/monitored (logins, logfile analysis, etc.). Use/adoption metrics are important process outcomes that should be reported in any ehealth trial.

	1	2	3	4	5	
subitem not at all important	○	○	○	○	○	essential

Does your paper address subitem 6a-ii?

Copy and paste relevant sections from manuscript text

Your answer

6a-iii) Describe whether, how, and when qualitative feedback from participants was obtained

Describe whether, how, and when qualitative feedback from participants was obtained (e.g., through emails, feedback forms, interviews, focus groups).

	1	2	3	4	5	
subitem not at all important	○	○	○	○	○	essential

Does your paper address subitem 6a-iii?

Copy and paste relevant sections from manuscript text

Your answer

6b) Any changes to trial outcomes after the trial commenced, with reasons

Does your paper address CONSORT subitem 6b? *

Copy and paste relevant sections from the manuscript (include quotes in quotation marks "like this" to indicate direct quotes from your manuscript), or elaborate on this item by providing additional information not in the ms, or briefly explain why the item is not applicable/relevant for your study

The trial was underpowered as recruitment had to be discontinued due to the COVID-19 pandemic: "A main limitation of this study is the small sample size, which was an unanticipated change due to the COVID-19 pandemic that resulted in discontinuing data collection in March 2020."

7a) How sample size was determined

NPT: When applicable, details of whether and how the clustering by care provides or centers was addressed

7a-i) Describe whether and how expected attrition was taken into account when calculating the sample size

Describe whether and how expected attrition was taken into account when calculating the sample size.

	1	2	3	4	5	
subitem not at all important	○	○	○	○	○	essential

?

Does your paper address subitem 7a-i?

Copy and paste relevant sections from manuscript title (include quotes in quotation marks "like this" to indicate direct quotes from your manuscript), or elaborate on this item by providing additional information not in the ms, or briefly explain why the item is not applicable/relevant for your study

Your answer

7b) When applicable, explanation of any interim analyses and stopping guidelines

Does your paper address CONSORT subitem 7b? *

Copy and paste relevant sections from the manuscript (include quotes in quotation marks "like this" to indicate direct quotes from your manuscript), or elaborate on this item by providing additional information not in the ms, or briefly explain why the item is not applicable/relevant for your study

Not applicable; interim analyses were not conducted.

8a) Method used to generate the random allocation sequence

NPT: When applicable, how care providers were allocated to each trial group

Does your paper address CONSORT subitem 8a? *

Copy and paste relevant sections from the manuscript (include quotes in quotation marks "like this" to indicate direct quotes from your manuscript), or elaborate on this item by providing additional information not in the ms, or briefly explain why the item is not applicable/relevant for your study

"Participants were then randomized to the intervention (food-specific) or control GNG task designed on the PsychoPy software (Open Science Tools Ltd) [33] using a computerized random assignment."

8b) Type of randomisation; details of any restriction (such as blocking and block size)

Does your paper address CONSORT subitem 8b? *

Copy and paste relevant sections from the manuscript (include quotes in quotation marks "like this" to indicate direct quotes from your manuscript), or elaborate on this item by providing additional information not in the ms, or briefly explain why the item is not applicable/relevant for your study

Not applicable; this was a pilot and feasibility randomized trial.

9) Mechanism used to implement the random allocation sequence (such as sequentially numbered containers), describing any steps taken to conceal the sequence until interventions were assigned

Does your paper address CONSORT subitem 9? *

Copy and paste relevant sections from the manuscript (include quotes in quotation marks "like this" to indicate direct quotes from your manuscript), or elaborate on this item by providing additional information not in the ms, or briefly explain why the item is not applicable/relevant for your study

A computerized random assignment was used.

10) Who generated the random allocation sequence, who enrolled participants, and who assigned participants to interventions

Does your paper address CONSORT subitem 10? *

Copy and paste relevant sections from the manuscript (include quotes in quotation marks "like this" to indicate direct quotes from your manuscript), or elaborate on this item by providing additional information not in the ms, or briefly explain why the item is not applicable/relevant for your study

"Participants were then randomized to the intervention (food-specific) or control GNG task designed on the PsychoPy software (Open Science Tools Ltd) [33] using a computerized random assignment. Following the GNG task, procedures took place in the following order at the laboratory visit: completion of hedonic appetite self-report measure, bogus taste test, self-report survey battery, eye tracking task, Automated Self-Administered 24-Hour Dietary Assessment Tool (ASA24) dietary recall, National Institutes of Health (NIH) Toolbox cognitive tasks, and anthropometric measurements. The research assistant responsible for assessing the outcome in the bogus taste test remained masked to the randomization condition so as not to introduce bias in outcome measurement. The research assistant administering the GNG task and eye tracking task was aware of the randomization group to administer the designated task."

11a) If done, who was blinded after assignment to interventions (for example, participants, care providers, those assessing outcomes) and how

NPT: Whether or not administering co-interventions were blinded to group assignment

11a-i) Specify who was blinded, and who wasn’t

Specify who was blinded, and who wasn’t. Usually, in web-based trials it is not possible to blind the participants [1, 3] (this should be clearly acknowledged), but it may be possible to blind outcome assessors, those doing data analysis or those administering co-interventions (if any).

	1	2	3	4	5	
subitem not at all important	○	○	○	○	○	essential

Does your paper address subitem 11a-i? *

Copy and paste relevant sections from the manuscript (include quotes in quotation marks "like this" to indicate direct quotes from your manuscript), or elaborate on this item by providing additional information not in the ms, or briefly explain why the item is not applicable/relevant for your study

The participants were blinded to their assignment and to the fact that this was a trial of 2 groups. The research coordinator administering the GNG task was aware of the assignment and this was intentionally a different research assistant than the one assessing the outcomes: "The research assistant responsible for assessing the outcome in the bogus taste test remained masked to the randomization condition so as not to introduce bias in outcome measurement. The research assistant administering the GNG task and eye tracking task was aware of the randomization group to administer the designated task."

11a-ii) Discuss e.g., whether participants knew which intervention was the “intervention of interest” and which one was the “comparator”

Informed consent procedures (4a-ii) can create biases and certain expectations - discuss e.g., whether participants knew which intervention was the “intervention of interest” and which one was the “comparator”.

	1	2	3	4	5	
subitem not at all important	○	○	○	○	○	essential

Does your paper address subitem 11a-ii?

Copy and paste relevant sections from the manuscript (include quotes in quotation marks "like this" to indicate direct quotes from your manuscript), or elaborate on this item by providing additional information not in the ms, or briefly explain why the item is not applicable/relevant for your study

Your answer

11b) If relevant, description of the similarity of interventions

(this item is usually not relevant for ehealth trials as it refers to similarity of a placebo or sham intervention to a active medication/intervention)

Does your paper address CONSORT subitem 11b? *

Copy and paste relevant sections from the manuscript (include quotes in quotation marks "like this" to indicate direct quotes from your manuscript), or elaborate on this item by providing additional information not in the ms, or briefly explain why the item is not applicable/relevant for your study

"The GNG procedures were designed in accordance with previous studies [25,34], including a practice round of the task to ensure that participants could perform it correctly. The GNG task included instructions to press a button when a go cue was presented and to not press a button when a no go cue was presented. Participants in the food-specific GNG intervention condition were trained using pictures of palatable foods grouped as sweet, starchy, fatty, and fast food as no go cues, with 4 food stimuli pictures used for each category (16 in total) and 16 nonfood stimuli pictures of animals as go cues. Those in the control GNG condition completed the GNG task with all nonfood stimuli (eg, animals and shapes; Figure 1). The images in the GNG tasks were used with approval from the Food-pics image database designed to facilitate standardization and comparability across studies [35] (Multimedia Appendix 1). For both groups, each stimulus was presented for 1000 ms, and the intertrial interval was 500 ms. If the participant made an incorrect response or did not respond within 1000 ms, a red cross appeared on the screen for 500 ms (Figure 1). For both groups, 50% of the trials were go trials (ie, 16 images of animals presented 4 times each), and 50% were no go trials."

12a) Statistical methods used to compare groups for primary and secondary outcomes

NPT: When applicable, details of whether and how the clustering by care providers or centers was addressed

Does your paper address CONSORT subitem 12a? *

Copy and paste relevant sections from the manuscript (include quotes in quotation marks "like this" to indicate direct quotes from your manuscript), or elaborate on this item by providing additional information not in the ms, or briefly explain why the item is not applicable/relevant for your study

"Effect of GNG Intervention
A series of independent-sample t tests were run to determine the effects of the GNG intervention versus the GNG control on gaze fixation on food images as measured using the eye tracker. Effects on hedonic appetite, palatable food consumption in the laboratory bogus taste test, and food consumption via dietary recall (second administration 3 days following the laboratory visit) were also examined."

12a-i) Imputation techniques to deal with attrition / missing values

Imputation techniques to deal with attrition / missing values: Not all participants will use the intervention/comparator as intended and attrition is typically high in ehealth trials. Specify how participants who did not use the application or dropped out from the trial were treated in the statistical analysis (a complete case analysis is strongly discouraged, and simple imputation techniques such as LOCF may also be problematic [4]).

	1	2	3	4	5	
subitem not at all important	○	○	○	○	○	essential

Does your paper address subitem 12a-i? *

Copy and paste relevant sections from the manuscript (include quotes in quotation marks "like this" to indicate direct quotes from your manuscript), or elaborate on this item by providing additional information not in the ms, or briefly explain why the item is not applicable/relevant for your study

We did not have a missing data issue for the outcomes of interest.

12b) Methods for additional analyses, such as subgroup analyses and adjusted analyses

Does your paper address CONSORT subitem 12b? *

Copy and paste relevant sections from the manuscript (include quotes in quotation marks "like this" to indicate direct quotes from your manuscript), or elaborate on this item by providing additional information not in the ms, or briefly explain why the item is not applicable/relevant for your study

"Preliminary Analyses
Bivariate correlations and independent-sample t tests were run to examine associations between potential covariates (eg, eating disorder symptomology and BMI percentile) and the dependent variables. Significant correlations and associations were then examined further using independent-sample t tests to examine potential differences between the participants randomized to the food GNG intervention group and the control GNG group, with each potential covariate as a dependent variable. In particular, the dietary intake data obtained through the dietary recall were used as a randomization check to determine whether the 2 groups ate significantly different amounts of total calories over the 24-hour period before the study visit. Information regarding participants' mental health and medication use that could potentially affect appetite and eating behavior was also examined as potential covariates. Information was coded as having a potential influence on dietary behavior or not, and chi-square analyses were run to determine any differences between the randomization groups.
Associations Between Inhibitory Control and Dietary Constructs
Regression models were run to examine cross-sectional relationships between inhibitory control as measured using the NIH Toolbox and the dietary constructs of interest (hedonic appetite, palatable food consumption in the laboratory, food consumption via dietary recall [first administration at the laboratory visit], and EPSI binge eating and cognitive restraint subscales)."

X26) REB/IRB Approval and Ethical Considerations [recommended as subheading under "Methods"] (not a CONSORT item)

X26-i) Comment on ethics committee approval

	1	2	3	4	5	
subitem not at all important	○	○	○	○	○	essential

Does your paper address subitem X26-i?

Copy and paste relevant sections from the manuscript (include quotes in quotation marks "like this" to indicate direct quotes from your manuscript), or elaborate on this item by providing additional information not in the ms, or briefly explain why the item is not applicable/relevant for your study

Your answer

x26-ii) Outline informed consent procedures

Outline informed consent procedures e.g., if consent was obtained offline or online (how? Checkbox, etc.?), and what information was provided (see 4a-ii). See [6] for some items to be included in informed consent documents.

	1	2	3	4	5	
subitem not at all important	○	○	○	○	○	essential

Does your paper address subitem X26-ii?

Copy and paste relevant sections from the manuscript (include quotes in quotation marks "like this" to indicate direct quotes from your manuscript), or elaborate on this item by providing additional information not in the ms, or briefly explain why the item is not applicable/relevant for your study

Your answer

X26-iii) Safety and security procedures

Safety and security procedures, incl. privacy considerations, and any steps taken to reduce the likelihood or detection of harm (e.g., education and training, availability of a hotline)

	1	2	3	4	5	
subitem not at all important	○	○	○	○	○	essential

Does your paper address subitem X26-iii?

Copy and paste relevant sections from the manuscript (include quotes in quotation marks "like this" to indicate direct quotes from your manuscript), or elaborate on this item by providing additional information not in the ms, or briefly explain why the item is not applicable/relevant for your study

Your answer

RESULTS

13a) For each group, the numbers of participants who were randomly assigned, received intended treatment, and were analysed for the primary outcome

NPT: The number of care providers or centers performing the intervention in each group and the number of patients treated by each care provider in each center

Does your paper address CONSORT subitem 13a? *

Copy and paste relevant sections from the manuscript (include quotes in quotation marks "like this" to indicate direct quotes from your manuscript), or elaborate on this item by providing additional information not in the ms, or briefly explain why the item is not applicable/relevant for your study

Yes, see Table 1: 43 participants with 21 Intervention and 22 control

13b) For each group, losses and exclusions after randomisation, together with reasons

Does your paper address CONSORT subitem 13b? (NOTE: Preferably, this is shown in a CONSORT flow diagram) *

Copy and paste relevant sections from the manuscript (include quotes in quotation marks "like this" to indicate direct quotes from your manuscript), or elaborate on this item by providing additional information not in the ms, or briefly explain why the item is not applicable/relevant for your study

There were no losses or exclusions after randomization

13b-i) Attrition diagram

Strongly recommended: An attrition diagram (e.g., proportion of participants still logging in or using the intervention/comparator in each group plotted over time, similar to a survival curve) or other figures or tables demonstrating usage/dose/engagement.

	1	2	3	4	5	
subitem not at all important	○	○	○	○	○	essential

Does your paper address subitem 13b-i?

Copy and paste relevant sections from the manuscript or cite the figure number if applicable (include quotes in quotation marks "like this" to indicate direct quotes from your manuscript), or elaborate on this item by providing additional information not in the ms, or briefly explain why the item is not applicable/relevant for your study

Your answer

14a) Dates defining the periods of recruitment and follow-up

Does your paper address CONSORT subitem 14a? *

Copy and paste relevant sections from the manuscript (include quotes in quotation marks "like this" to indicate direct quotes from your manuscript), or elaborate on this item by providing additional information not in the ms, or briefly explain why the item is not applicable/relevant for your study

Please see CONSORT diagram

14a-i) Indicate if critical “secular events” fell into the study period

Indicate if critical “secular events” fell into the study period, e.g., significant changes in Internet resources available or “changes in computer hardware or Internet delivery resources”

	1	2	3	4	5	
subitem not at all important	○	○	○	○	○	essential

Does your paper address subitem 14a-i?

Copy and paste relevant sections from the manuscript (include quotes in quotation marks "like this" to indicate direct quotes from your manuscript), or elaborate on this item by providing additional information not in the ms, or briefly explain why the item is not applicable/relevant for your study

Your answer

14b) Why the trial ended or was stopped (early)

Does your paper address CONSORT subitem 14b? *

Copy and paste relevant sections from the manuscript (include quotes in quotation marks "like this" to indicate direct quotes from your manuscript), or elaborate on this item by providing additional information not in the ms, or briefly explain why the item is not applicable/relevant for your study

""A main limitation of this study is the small sample size, which was an unanticipated change due to the COVID-19 pandemic that resulted in discontinuing data collection in March 2020."

15) A table showing baseline demographic and clinical characteristics for each group

NPT: When applicable, a description of care providers (case volume, qualification, expertise, etc.) and centers (volume) in each group

Does your paper address CONSORT subitem 15? *

Copy and paste relevant sections from the manuscript (include quotes in quotation marks "like this" to indicate direct quotes from your manuscript), or elaborate on this item by providing additional information not in the ms, or briefly explain why the item is not applicable/relevant for your study

Please see Table 1

15-i) Report demographics associated with digital divide issues

In ehealth trials it is particularly important to report demographics associated with digital divide issues, such as age, education, gender, social-economic status, computer/Internet/ehealth literacy of the participants, if known.

	1	2	3	4	5	
subitem not at all important	○	○	○	○	○	essential

Does your paper address subitem 15-i? *

Copy and paste relevant sections from the manuscript (include quotes in quotation marks "like this" to indicate direct quotes from your manuscript), or elaborate on this item by providing additional information not in the ms, or briefly explain why the item is not applicable/relevant for your study

Please see Table 1

16) For each group, number of participants (denominator) included in each analysis and whether the analysis was by original assigned groups

16-i) Report multiple "denominators" and provide definitions

Report multiple "denominators" and provide definitions: Report N's (and effect sizes) "across a range of study participation [and use] thresholds" [1], e.g., N exposed, N consented, N used more than x times, N used more than y weeks, N participants "used" the intervention/comparator at specific pre-defined time points of interest (in absolute and relative numbers per group). Always clearly define "use" of the intervention.

	1	2	3	4	5	
subitem not at all important	○	○	○	○	○	essential

Does your paper address subitem 16-i? *

Copy and paste relevant sections from the manuscript (include quotes in quotation marks "like this" to indicate direct quotes from your manuscript), or elaborate on this item by providing additional information not in the ms, or briefly explain why the item is not applicable/relevant for your study

Please see Table 1

16-ii) Primary analysis should be intent-to-treat

Primary analysis should be intent-to-treat, secondary analyses could include comparing only “users”, with the appropriate caveats that this is no longer a randomized sample (see 18-i).

	1	2	3	4	5	
subitem not at all important	○	○	○	○	○	essential

Does your paper address subitem 16-ii?

Copy and paste relevant sections from the manuscript (include quotes in quotation marks "like this" to indicate direct quotes from your manuscript), or elaborate on this item by providing additional information not in the ms, or briefly explain why the item is not applicable/relevant for your study

Your answer

17a) For each primary and secondary outcome, results for each group, and the estimated effect size and its precision (such as 95% confidence interval)

Does your paper address CONSORT subitem 17a? *

Copy and paste relevant sections from the manuscript (include quotes in quotation marks "like this" to indicate direct quotes from your manuscript), or elaborate on this item by providing additional information not in the ms, or briefly explain why the item is not applicable/relevant for your study

"In the small subsample of participants who completed the gaze fixation task with eye tracking (15/43, 34.9%), participants from the intervention condition looked at food images for 20.85 (SD=10.25) seconds and participants from the control group looked at food images for 23.01 (SD=8.39) seconds. Therefore, the participants from the intervention condition looked at food images for 2.1 seconds less than control participants in absolute terms (95% CI −8.3 to 12.6; Table 2). Expressed as an effect size, this is considered a small effect (Cohen d=0.23). There were no other significant differences in hedonic appetite or food consumption via the bogus taste test or dietary recall following the laboratory visit. Given the limited subsample, these are considered preliminary findings that should be examined in a fully powered study."

17a-i) Presentation of process outcomes such as metrics of use and intensity of use

In addition to primary/secondary (clinical) outcomes, the presentation of process outcomes such as metrics of use and intensity of use (dose, exposure) and their operational definitions is critical. This does not only refer to metrics of attrition (13-b) (often a binary variable), but also to more continuous exposure metrics such as "average session length". These must be accompanied by a technical description how a metric like a "session" is defined (e.g., timeout after idle time) [1] (report under item 6a).

	1	2	3	4	5	
subitem not at all important	○	○	○	○	○	essential

Does your paper address subitem 17a-i?

Copy and paste relevant sections from the manuscript (include quotes in quotation marks "like this" to indicate direct quotes from your manuscript), or elaborate on this item by providing additional information not in the ms, or briefly explain why the item is not applicable/relevant for your study

Your answer

17b) For binary outcomes, presentation of both absolute and relative effect sizes is recommended

Does your paper address CONSORT subitem 17b? *

Copy and paste relevant sections from the manuscript (include quotes in quotation marks "like this" to indicate direct quotes from your manuscript), or elaborate on this item by providing additional information not in the ms, or briefly explain why the item is not applicable/relevant for your study

Not applicable; no binary outcomes

18) Results of any other analyses performed, including subgroup analyses and adjusted analyses, distinguishing pre-specified from exploratory

Does your paper address CONSORT subitem 18? *

Copy and paste relevant sections from the manuscript (include quotes in quotation marks "like this" to indicate direct quotes from your manuscript), or elaborate on this item by providing additional information not in the ms, or briefly explain why the item is not applicable/relevant for your study

"In the small subsample of participants who completed the NIH Toolbox flanker task to measure inhibitory control (19/43, 44.2%), inhibitory control was significantly and positively associated with sugar intake as measured through the laboratory-based administration of the dietary recall ($F_{10}=18.5$; $P<.01$), with an effect size ($\eta 2$) of 0.979 (95% CI 0.43-0.97). Inhibitory control was also marginally related to binge eating ($F(13)=4.53$; $P=.05$; $\eta 2=0.922$, 95% CI 0.00-0.89) and cognitive restraint ($F(13)=4.05$; $P=.06$; $\eta 2=0.913$, 95% CI 0.00-0.87) as measured using the EPSI. There were no significant associations or trends between inhibitory control and hedonic appetite or food consumption at the laboratory visit. It must be noted that this is a limited subsample and the associations presented do not meet conventional significance thresholds. These findings represent preliminary patterns that should be investigated in fully powered studies."

18-i) Subgroup analysis of comparing only users

A subgroup analysis of comparing only users is not uncommon in ehealth trials, but if done, it must be stressed that this is a self-selected sample and no longer an unbiased sample from a randomized trial (see 16-iii).

	1	2	3	4	5	
subitem not at all important	○	○	○	○	○	essential

Does your paper address subitem 18-i?

Copy and paste relevant sections from the manuscript (include quotes in quotation marks "like this" to indicate direct quotes from your manuscript), or elaborate on this item by providing additional information not in the ms, or briefly explain why the item is not applicable/relevant for your study

Your answer

19) All important harms or unintended effects in each group

(for specific guidance see CONSORT for harms)

Does your paper address CONSORT subitem 19? *

Copy and paste relevant sections from the manuscript (include quotes in quotation marks "like this" to indicate direct quotes from your manuscript), or elaborate on this item by providing additional information not in the ms, or briefly explain why the item is not applicable/relevant for your study

Not applicable; no harms or unintended effects.

19-i) Include privacy breaches, technical problems

Include privacy breaches, technical problems. This does not only include physical “harm” to participants, but also incidents such as perceived or real privacy breaches [1], technical problems, and other unexpected/unintended incidents. “Unintended effects” also includes unintended positive effects [2].

	1	2	3	4	5	
subitem not at all important	○	○	○	○	○	essential

Does your paper address subitem 19-i?

Copy and paste relevant sections from the manuscript (include quotes in quotation marks "like this" to indicate direct quotes from your manuscript), or elaborate on this item by providing additional information not in the ms, or briefly explain why the item is not applicable/relevant for your study

Your answer

19-ii) Include qualitative feedback from participants or observations from staff/researchers

Include qualitative feedback from participants or observations from staff/researchers, if available, on strengths and shortcomings of the application, especially if they point to unintended/unexpected effects or uses. This includes (if available) reasons for why people did or did not use the application as intended by the developers.

	1	2	3	4	5	
subitem not at all important	○	○	○	○	○	essential

Does your paper address subitem 19-ii?

Copy and paste relevant sections from the manuscript (include quotes in quotation marks "like this" to indicate direct quotes from your manuscript), or elaborate on this item by providing additional information not in the ms, or briefly explain why the item is not applicable/relevant for your study

Your answer

DISCUSSION

22) Interpretation consistent with results, balancing benefits and harms, and considering other relevant evidence

NPT: In addition, take into account the choice of the comparator, lack of or partial blinding, and unequal expertise of care providers or centers in each group

22-i) Restate study questions and summarize the answers suggested by the data, starting with primary outcomes and process outcomes (use)

Restate study questions and summarize the answers suggested by the data, starting with primary outcomes and process outcomes (use).

	1	2	3	4	5	
subitem not at all important	○	○	○	○	○	essential

Does your paper address subitem 22-i? *

Copy and paste relevant sections from the manuscript (include quotes in quotation marks "like this" to indicate direct quotes from your manuscript), or elaborate on this item by providing additional information not in the ms, or briefly explain why the item is not applicable/relevant for your study

"Principal Findings
This study found that the food-specific GNG ICT intervention had preliminary associations with attentional bias toward food cues as measured through an eye tracking gaze fixation task compared to a control GNG ICT task. Additionally, inhibitory control as measured using the NIH Toolbox was significantly associated with sugar intake as measured using the dietary recall completed at the laboratory visit and showed marginally significant cross-sectional associations with binge eating and cognitive restraint. Our findings contribute to the literature examining the mechanisms through which ICT functions. Understanding of these mechanisms is still in process in the field, with a few possible pathways, including the devaluation processes [47]. This means that attention is often allocated toward rewarding palatable food stimuli; therefore, decreasing the reward value of palatable food stimuli is a possible mechanism that could be at the core of these processes. Still, attentional bias has had mixed associations with palatable food intake thus far [5,48,49]. Further research is needed to examine the role of attention allocation in predicting eating behavior in adolescents and whether ICT is the most effective intervention.
Contrary to the hypotheses, the GNG intervention did not have any effect on hedonic appetite or food consumption measured either in the laboratory or via the dietary recall, and inhibitory control was not related to hedonic appetite or food consumption in the laboratory visit. While this study was indeed limited by the small sample size, we note that there are some mixed associations between inhibitory control and eating behavior in adolescents in the literature, with some studies showing no association [50] and others showing low inhibitory control as a risk factor for developing disordered eating [51]. While a large portion of the literature has focused on inhibitory control–binge eating relationships in the context of obesity, inhibitory control difficulties have been linked to loss-of-control eating in community samples of adolescents as well [2]. Interestingly, in our sample, inhibitory control was related to sugar consumption and binge eating in the opposite direction than hypothesized. In this small sample, higher inhibitory control was associated with higher sugar consumption and risk of binge eating. Considering this unexpected association and the fact that inhibitory control was also marginally associated with cognitive restraint, the mechanistic role of inhibitory control should be examined in fully powered studies aligned with the neurobehavioral inhibitory control model [7]."

22-ii) Highlight unanswered new questions, suggest future research

Highlight unanswered new questions, suggest future research.

	1	2	3	4	5	
subitem not at all important	○	○	○	○	○	essential

Does your paper address subitem 22-ii?

Copy and paste relevant sections from the manuscript (include quotes in quotation marks "like this" to indicate direct quotes from your manuscript), or elaborate on this item by providing additional information not in the ms, or briefly explain why the item is not applicable/relevant for your study

Your answer

20) Trial limitations, addressing sources of potential bias, imprecision, and, if relevant, multiplicity of analyses

20-i) Typical limitations in ehealth trials

Typical limitations in ehealth trials: Participants in ehealth trials are rarely blinded. Ehealth trials often look at a multiplicity of outcomes, increasing risk for a Type I error. Discuss biases due to non-use of the intervention/usability issues, biases through informed consent procedures, unexpected events.

	1	2	3	4	5	
subitem not at all important	○	○	○	○	○	essential

Does your paper address subitem 20-i? *

Copy and paste relevant sections from the manuscript (include quotes in quotation marks "like this" to indicate direct quotes from your manuscript), or elaborate on this item by providing additional information not in the ms, or briefly explain why the item is not applicable/relevant for your study

"A main limitation of this study is the small sample size, which was an unanticipated change due to the COVID-19 pandemic that resulted in discontinuing data collection in March 2020. This study is also limited by administration of the eye tracking task and the NIH Toolbox in subsamples of our total sample. This was done to pilot each methodology with the adolescent age group and as part of the laboratory visit procedures overall. Because only a subsample completed the eye tracking and NIH Toolbox tasks, we cannot make any claims about external validity. It must be noted that these small subsamples limit power and generalizability and cannot be taken as definitive. The findings should be considered preliminary, and these procedures should be conducted in fully powered studies as a next step."

21) Generalisability (external validity, applicability) of the trial findings

NPT: External validity of the trial findings according to the intervention, comparators, patients, and care providers or centers involved in the trial

21-i) Generalizability to other populations

Generalizability to other populations: In particular, discuss generalizability to a general Internet population, outside of a RCT setting, and general patient population, including applicability of the study results for other organizations

	1	2	3	4	5	
subitem not at all important	○	○	○	○	○	essential

Does your paper address subitem 21-i?

Copy and paste relevant sections from the manuscript (include quotes in quotation marks "like this" to indicate direct quotes from your manuscript), or elaborate on this item by providing additional information not in the ms, or briefly explain why the item is not applicable/relevant for your study

"Additionally, participants in this study were predominantly White, female, and from higher-income households. Recruiting more demographically diverse individuals is a necessary focus for future research in this area, especially as systemic inequities based on race and ethnicity, socioeconomic status (eg, food insecurity), and cultural factors influence eating behavior and overall health [52]. The lack of diversity in this study also limits the applicability of the results to broader adolescent populations, which can be improved upon in larger, fully powered, and representative studies."

21-ii) Discuss if there were elements in the RCT that would be different in a routine application setting

Discuss if there were elements in the RCT that would be different in a routine application setting (e.g., prompts/reminders, more human involvement, training sessions or other co-interventions) and what impact the omission of these elements could have on use, adoption, or outcomes if the intervention is applied outside of a RCT setting.

	1	2	3	4	5	
subitem not at all important	○	○	○	○	○	essential

Does your paper address subitem 21-ii?

Copy and paste relevant sections from the manuscript (include quotes in quotation marks "like this" to indicate direct quotes from your manuscript), or elaborate on this item by providing additional information not in the ms, or briefly explain why the item is not applicable/relevant for your study

Your answer

OTHER INFORMATION

23) Registration number and name of trial registry

Does your paper address CONSORT subitem 23? *

Copy and paste relevant sections from the manuscript (include quotes in quotation marks "like this" to indicate direct quotes from your manuscript), or elaborate on this item by providing additional information not in the ms, or briefly explain why the item is not applicable/relevant for your study

Not applicable; this is a pilot and feasibility randomized controlled trial. Confirmed with managing editor that this is not needed.

24) Where the full trial protocol can be accessed, if available

Does your paper address CONSORT subitem 24? *

Cite a Multimedia Appendix, other reference, or copy and paste relevant sections from the manuscript (include quotes in quotation marks "like this" to indicate direct quotes from your manuscript), or elaborate on this item by providing additional information not in the ms, or briefly explain why the item is not applicable/relevant for your study

Not applicable; this is a pilot and feasibility randomized controlled trial. Confirmed with managing editor that this is not needed.

25) Sources of funding and other support (such as supply of drugs), role of funders

Does your paper address CONSORT subitem 25? *

Copy and paste relevant sections from the manuscript (include quotes in quotation marks "like this" to indicate direct quotes from your manuscript), or elaborate on this item by providing additional information not in the ms, or briefly explain why the item is not applicable/relevant for your study

"This study was funded by the Society of Pediatric Psychology Marion and Donald Routh Student Research Grant and the University of Kansas Office of Graduate Studies Doctoral Student Research Fund awarded to CB under the mentorship of CC. AO is supported by a grant from the National Institutes of Health. The funders had no role in the design and conduct of the study; collection, management, analysis, and interpretation of the data; preparation, review, or approval of the manuscript; or decision to submit the manuscript for publication."

X27) Conflicts of Interest (not a CONSORT item)

X27-i) State the relation of the study team towards the system being evaluated

In addition to the usual declaration of interests (financial or otherwise), also state the relation of the study team towards the system being evaluated, i.e., state if the authors/evaluators are distinct from or identical with the developers/sponsors of the intervention.

	1	2	3	4	5	
subitem not at all important	○	○	○	○	○	essential

Does your paper address subitem X27-i?

Copy and paste relevant sections from the manuscript (include quotes in quotation marks "like this" to indicate direct quotes from your manuscript), or elaborate on this item by providing additional information not in the ms, or briefly explain why the item is not applicable/relevant for your study

Your answer

About the CONSORT EHEALTH checklist

As a result of using this checklist, did you make changes in your manuscript? *

- ( ) yes, major changes
- ( ) yes, minor changes
- (•) no

What were the most important changes you made as a result of using this checklist?

Your answer

How much time did you spend on going through the checklist INCLUDING making changes in your manuscript *

2 hours

As a result of using this checklist, do you think your manuscript has improved? *

- ( ) yes
- (•) no
- ( ) Other:

Would you like to become involved in the CONSORT EHEALTH group?

This would involve for example becoming involved in participating in a workshop and writing an "Explanation and Elaboration" document

- ○ yes
- ○ no
- ○ Other:

Any other comments or questions on CONSORT EHEALTH

Your answer

STOP - Save this form as PDF before you click submit

To generate a record that you filled in this form, we recommend to generate a PDF of this page (on a Mac, simply select "print" and then select "print as PDF") before you submit it.

When you submit your (revised) paper to JMIR, please upload the PDF as supplementary file.

Don't worry if some text in the textboxes is cut off, as we still have the complete information in our database. Thank you!

Final step: Click submit !

Click submit so we have your answers in our database!

Submit Clear form

Never submit passwords through Google Forms.

This content is neither created nor endorsed by Google. - Contact form owner - Terms of Service - Privacy Policy

Does this form look suspicious? Report

Google Forms
